# International Trends in Technological Progress: Evidence from Patent Citations, 1980–2011

**DOI:** 10.1111/ecoj.12314

**Published:** 2017-10-24

**Authors:** Soonwoo Kwon, Jihong Lee, Sokbae Lee

**Affiliations:** ^1^ Yale University; ^2^ Seoul National University; ^3^ Centre for Microdata Methods and Practice Institute for Fiscal Studies

## Abstract

We analyse cross‐country trends in several aspects of technological progress during 1980–2011 by examining the US patent citations data. Our estimation results on patent quality and citation lags relative to the US reveal the following. The emerging Asian economies of Korea, Taiwan and China have achieved substantial catch‐up. In the case of Korea and Taiwan, progress has been made in both patent quality and citation lags. China has achieved improvement in patent quality but not in citation lag. In contrast, advanced economies of Europe and Japan have displayed steady decline in patent quality, while the US has strengthened its position.

This article documents comprehensive cross‐country trends in several aspects of technological progress over the period of 1980–2011 by examining a newly updated citations dataset from almost 4 million utility patents granted by the US Patent and Trademark Office (USPTO). The purpose of our study is to provide a rigorous international comparison that reflects underlying innovation qualities and help better understand the substance behind rapidly rising volumes of patents granted by major patent offices in recent years.^1^

Since the study of Trajtenberg (1990), citations data have offered an important source of patent quality measure: higher quality patents should generate more impact and hence more citations. The first part of our analysis studies the patent quality of different countries as measured by ‘average citations’ received within two years.^2^ Specifically, we develop a panel regression model with fixed effects to compare the citation rates of non‐US inventors to that of US inventors in each of the three decades, 1980s, 1990s and 2000s. We overcome the issue of country‐wide heterogeneity by focusing on USPTO‐granted patents, and address the time‐varying nature of USPTO practices by adopting the method of difference‐in‐differences.^3^

Our regression results yield several noteworthy findings. First, patent quality of the emerging Asian economies of Korea and Taiwan rapidly caught up with the US during the 1990s, while similar catch‐up occurred for China during the 2000s. Therefore, our results suggest that the recent surge of Korea, Taiwan and China in the volume of patent production could also be substantiated by underlying quality improvement. In contrast, the advanced nations of Europe and Japan have experienced a steady decline in their patent quality. While they too produce substantially more patents now than before, when measured *vis‐à‐vis* the US, patent quality of these countries has fallen across each of the three sample decades. The US has, on the other hand, strengthened its position in the international patent quality ladder despite the inroads made by a number of emerging economies.^4^

The patent citations data offer another avenue to obtain an output indicator of innovation activity by different countries. In the second part of our article, we take US‐inventors’ patents as the frontier technology and implement the fixed‐effects estimator of Griffith *et al*. (2011) to measure the ‘citation lags’, i.e. the speeds with which non‐US inventors cite the frontier patents relative to US‐inventors. We find that citation lags for the advanced countries of Europe and Japan *vis‐à‐vis* the US did not change significantly over the last three decades, while most significant gains in narrowing of the citation lag were achieved by the emerging economies of Korea, Taiwan and Israel. These observations strengthen the case for overall technological progress achieved by the latter group of countries. China, for whom our evidence on patent quality trends suggest significant upgrade, did not register a similar level of improvement on the citation lag.

Our article contributes above all to the large and extensive literature on investigating patents as economic indicators.^5^ Within this literature, an early related study on using patent citations as a quality indicator was by Trajtenberg (1990), who estimated the effect of various research inputs on the citation‐weighted patent counts.^6^ An index of patent quality using citations information was developed by Lanjouw and Schankerman (2004).

Another area in which patent citations have been used extensively is the literature on knowledge diffusion. Using patent citation information as a direct measure of the transfer of knowledge, this empirical literature explores the role of distance, e.g. national boundaries. See Jaffe *et al*. (1993), Jaffe and Trajtenberg (1999), Hu and Jaffe (2003), Henderson *et al*. (2005), Thompson and Fox‐Kean (2005), Thompson (2006) and Griffith *et al*. (2011), among many others. In contrast, this article uses citations from an updated dataset to track the recent cross‐country trends in technological progress.

The remainder of the article proceeds as follows. In Section 1, we describe our data. In Section 2, we study cross‐country trends in patent quality by employing differences‐in‐differences estimation against the US benchmark in different time periods. In Section 3, we measure distance to the knowledge frontier, using the fixed‐effects estimator of Griffith *et al*. (2011), and document the trends across countries. Section 4 offers some concluding remarks. Online Appendices provide further details on our dataset as well as additional analyses that are left out of the main text for expositional reasons.

## 1 Data

We use USPTO patent citation data from January 1980 to December 2011. The data up to 1999 are obtained from the National Bureau of Economic Research (NBER) US Patent Citations Data Files – specifically, PAT63_99 and CITE75_99.^7^ Bronwyn Hall provides the corresponding data for additional three years, over the period 2000–2, on her website.^8^ For this article, we have extended the dataset up to December 2011 by extracting related information (including the inventor location) from the bulk data provided by the USPTO. Online Appendix A presents the details of how this data extension was conducted. Combining the two previous sets of data with our data extracts, we constructed a dataset consisting of all utility patents granted up to December 2011 and detailed information on the patents that cited other patents included in the dataset. The number of patents that we have added for the period of 2003–11 amounts to over 1.5 million, equivalent to almost 40% of the sample size.

Our analysis considers the top 15 countries in terms of the accumulated number of utility patents granted by the USPTO between 1980 and 2011 (see Table 1).^9^ These 15 countries, ordered from the most granted to the least, are as follows: United States (US), Japan (JP), Germany (DE), EU,^10^ France (FR), United Kingdom (UK), Taiwan (TW), Korea (KR), Canada (CA), Switzerland (CH), Australia (AU), Israel (IL), China (CN),^11^ India (IN) and Former Soviet Union states (FSU).^12^ Our estimation analyses below also consider patents produced by the Rest of the World (RW).

**Table 1:** Patent Counts

	Period			
Country	1980–9	1990–9	2000–11	Total
United States (US)	393,595	602,865	1,053,089	2,049,549
Japan (JP)	122,402	237,093	431,503	790,998
Germany (DE)	66,517	75,085	125,312	266,914
EU	35,416	48,272	88,628	172,316
France (FR)	23,744	30,674	43,875	98,293
United Kingdom (UK)	24,556	27,188	43,447	95,191
Taiwan (TW)	2,170	17,638	74,349	94,157
Korea (KR)	522	14,256	75,880	90,658
Canada (CA)	13,110	22,727	43,974	79,811
Switzerland (CH)	12,268	11,927	15,540	39,735
Australia (AU)	3,398	4,984	14,008	22,390
Israel (IL)	1,797	4,501	14,626	20,924
China (CN)	139	571	11,825	12,535
India (IN)	108	442	6,321	6,871
Former Soviet Union (FSU)	2,119	1,442	2,925	6,486
Rest of the World (RW)	6,611	8,726	24,983	40,320
Total	708,472	1,108,391	2,070,285	3,887,148

*Notes*. Data consist of all utility patents granted at the United States Patent Office (USPTO) between 1980 and 2011. A country of a patent is classified by the location of the first inventor. EU refers to 15 European Union members as of 2002 except Germany, France and United Kingdom.

## 2 Patent Quality

### 2.1 Regression Results

The patent data offer a valuable source for exploring the innovation capacity of a country. In this subsection, we analyse cross‐country trends in patent quality measured by citations. Specifically, we develop a panel regression model with fixed effects to compare the patent quality of non‐US inventors to that of US inventors over the period of 1980–2011. Our model is given by

(1) $$Y_{cst}=\alpha_{0}+\Sigma_{i=1}^{36}\alpha_{i}s_{i}+\Sigma_{i=1}^{29}\beta_{i}t_{i}+\eta_{1}^{\prime }X_{cst,80}+\eta_{2}^{\prime }X_{cst,90}+\eta_{3}^{\prime }X_{cst,00}+\varepsilon_{cst},$$

where $Y_{cst}$ is the measure of the patent quality for country *c*, sector *s* and grant year *t*,^13^ $\left\{s_{i}\right\}$ are 36 sector (sub‐category) dummies,^14^ $\left\{t_{i}\right\}$ are 29 grant year dummies,^15^ and $\varepsilon_{cst}$ is the regression error term. In addition, the following regressors are included to obtain difference‐in‐differences (DiD) estimates (*vis‐à‐vis* the US):

$$\begin{array}{cc} X_{cst,80} & ={\left(1\left\{c=\text{JP},1980\leq t\leq 1989\right\},\ldots ,1\left\{c=\text{RW},1980\leq t\leq 1989\right\}\right)}^{\prime },\\ X_{cst,90} & ={\left(1\left\{c=\text{JP},1990\leq t\leq 1999\right\},\ldots ,1\left\{c=\text{RW},1990\leq t\leq 1999\right\}\right)}^{\prime },\\ X_{cst,00} & ={\left(1\left\{c=\text{JP},2000\leq t\leq 2009\right\},\ldots ,1\left\{c=\text{RW},2000\leq t\leq 2009\right\}\right)}^{\prime }, \end{array}$$

where **1**{·} is the usual indicator function.^16^ These regressors are vectors of indicator variables across (non‐US) countries for each of the three sample decades. For instance, the first element of $X_{cst,80}$ has value one only for Japan in the 1980s, while its *j*th element contains value one for the country reported in the (*j* + 1)‐th row of Table 1. $X_{cst,90}$ and $X_{cst,80}$ are defined similarly.

While there are potentially many alternative methods of measuring patent quality from citations data, one natural measure is to simply count the number of citations that a patent receives. We consider the average number of citations that a patent granted to country *c*, sector *s* and year *t* accumulates within the first two years from the grant date. Our main dependent variable is the logarithm of this measure of ‘average citations’.^17^

The citations measure of patent quality may however admit the effects of size and home biases. In particular, US patents may receive more citations because of the large overall US share of patents combined with the fact that our data come from USPTO. These biases may also be significant for countries that witnessed rapid rise in patent numbers during sample years.

To deal with this issue, we run a parallel regression on the logarithm of ‘adjusted average citations’ constructed as follows. We first compute the (two‐year) average citations excluding citations from the same country and sub‐category combination and then renormalise it by one minus that country/sub‐category's grant share. Specifically, for each (*c*,*s*,*t*), the adjusted average citations measure is defined as

$$\frac{\text{Average citations, excluding citations made by patents from}\;(c,s)}{1-\left(\#\;\text{of patents in the}\;(c,s,t)\;\text{cell}\right)/\left(\text{Total}\;\#\;\text{of patents in grant year}\;t\right)}.$$

Excluding citations from patents from the same country and sub‐category combination completely eliminates home bias but it requires some normalisation since one also needs to adjust for the size of the set of ‘potentially citing’ patents for each country/sub‐category/grant year cell. This latter issue is complicated by the fact that the patent share changes over time, and the citations come from all future years. Our normalisation divides the average citations measure by one minus the country/sub‐category's patent share in the given year.

The parameters of interest are the coefficients $\eta_{1}$, $\eta_{2}$ and $\eta_{3}$, which capture the difference compared to the US in the 1980s, 1990s and 2000s respectively for each country.^18^ To account for the different sample sizes of cohorts, we adopt weighted regression with the weights given by the number of patents in each cell. Standard errors are clustered by country and sub‐category.

**Figure 1:**
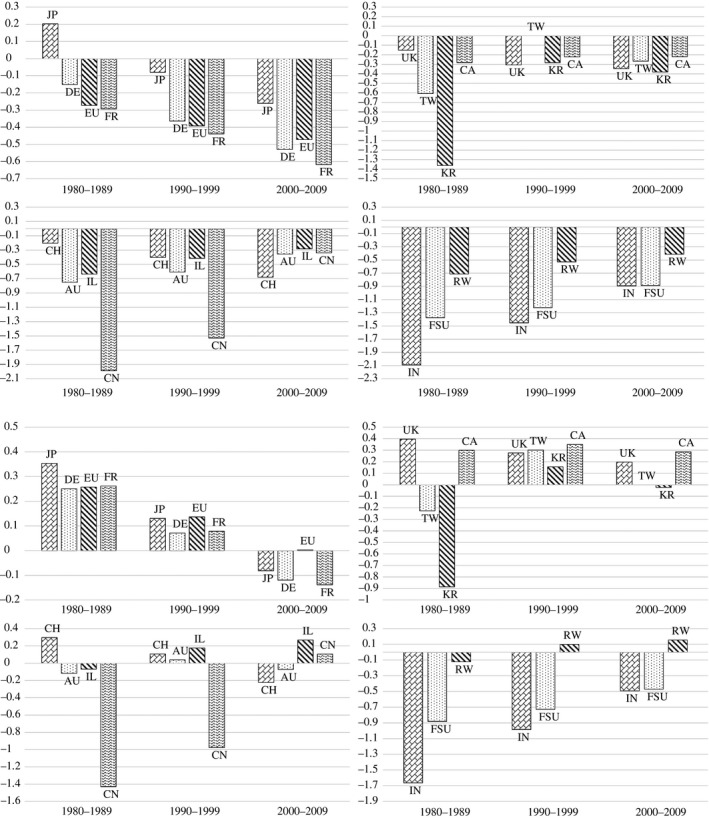
Graphical Representation of Estimation Results in Table 2 *Notes*. The top (bottom) four charts correspond to the coefficient values from the first (second) regression reported in Table 2.

Table 2 reports the estimation results on the sector‐adjusted and grant‐year‐adjusted cross‐country trends in patent quality represented by two different citation measures. The coefficient values are also illustrated via clustered bar charts in Figure 1.

**Table 2:** Regression Results (Log Average Citations)

	Dependent variable					
	Log average citations			Log average citations (adjusted)		
	(1)	(2)	(3)	(4)	(5)	(6)
Country	1980–9	1990–9	2000–9	1980–9	1990–9	2000–9
Japan (JP)	0.203***	−0.079	−0.260***	0.354***	0.131***	−0.081*
	(0.051)	(0.041)	(0.048)	(0.049)	(0.034)	(0.040)
Germany (DE)	−0.150**	−0.363***	−0.529***	0.251***	0.072	−0.119**
	(0.049)	(0.037)	(0.046)	(0.046)	(0.038)	(0.038)
EU	−0.272***	−0.391***	−0.471***	0.258***	0.137**	0.003
	(0.051)	(0.040)	(0.050)	(0.046)	(0.044)	(0.062)
France (FR)	−0.293***	−0.438***	−0.616***	0.262***	0.079	−0.138**
	(0.048)	(0.042)	(0.039)	(0.046)	(0.046)	(0.049)
United Kingdom (UK)	−0.151*	−0.304***	−0.344***	0.397***	0.277***	0.197***
	(0.067)	(0.049)	(0.044)	(0.056)	(0.044)	(0.060)
Taiwan (TW)	−0.605***	−0.000	−0.266**	−0.225**	0.302***	−0.000
	(0.080)	(0.082)	(0.086)	(0.071)	(0.057)	(0.062)
Korea (KR)	−1.359***	−0.282***	−0.380***	−0.885***	0.157	−0.022
	(0.142)	(0.069)	(0.052)	(0.155)	(0.081)	(0.057)
Canada (CA)	−0.281***	−0.221***	−0.218***	0.300***	0.351***	0.287***
	(0.051)	(0.046)	(0.044)	(0.049)	(0.045)	(0.060)
Switzerland (CH)	−0.202*	−0.399***	−0.678***	0.298***	0.107	−0.221***
	(0.084)	(0.056)	(0.058)	(0.078)	(0.063)	(0.069)
Australia (AU)	−0.747***	−0.605***	−0.352***	−0.116	0.039	−0.071
	(0.084)	(0.068)	(0.091)	(0.088)	(0.083)	(0.161)
Israel (IL)	−0.634***	−0.413***	−0.280***	−0.067	0.177	0.269**
	(0.109)	(0.088)	(0.077)	(0.105)	(0.104)	(0.087)
China (CN)	−1.985***	−1.529***	−0.338*	−1.427***	−0.975***	0.106
	(0.202)	(0.178)	(0.149)	(0.211)	(0.163)	(0.126)
India (IN)	−2.088***	−1.450***	−0.890***	−1.663***	−0.983***	−0.490*
	(0.230)	(0.164)	(0.148)	(0.184)	(0.187)	(0.209)
Former Soviet Union (FSU)	−1.373***	−1.220***	−0.886***	−0.880***	−0.725***	−0.469**
	(0.106)	(0.140)	(0.123)	(0.136)	(0.172)	(0.143)
Rest of the World (RW)	−0.711***	−0.526***	−0.410***	−0.120	0.100	0.156**
	(0.060)	(0.051)	(0.053)	(0.064)	(0.065)	(0.055)

*Notes*. Columns (1)–(3) show estimated coefficient values for the three DiD regressors with log average citations as the dependent variable, and columns (4)–(6) contain corresponding estimates for an adjusted version of the log average citations. *p < 0.05, **p < 0.01, ***p < 0.001. In the parentheses are standard errors. In each regression, grant year dummies and sub‐category dummies are also included as regressors. We use standard errors clustered by country and sub‐category. We use weighted regression where the sample size of each cohort is used as the weight. There are total 15,746 observations. In the first (second) regression, zero average citations are replaced by 0.018 (0.014) in 1,781 (1,972) observations.

We summarise our findings as follows:

Finding 1 Patent quality of the emerging Asian economies of Korea and Taiwan caught up with the US during the 1990s, while similar catch‐up occurred for China during the 2000s.

Patent quality of Korea and Taiwan both lagged substantially behind that of the US in the 1980s. In terms of (unadjusted) log average citations, the coefficient value of the DiD regressor was −1.359 for Korea (or roughly 74% below the US) and −0.605 (≈ −45%) for Taiwan, with both measures being significant at 0.1% level.^19^ The corresponding figures on adjusted log average citations were −0.885 and −0.225 respectively. This means that the adjusted average citations were around 59% and 20% lower for Korea and Taiwan respectively.

In the 1990s, however, this deficit in log average citations was reduced almost by a factor of 5 for Korea to −0.282, implying that the average citation of Korean patents had increased by about 50% relative to the US, while Taiwan achieved parity with the US. This rapid catch‐up was also apparent after adjusting for the home bias. The negative coefficients in adjusted log average citations for the two countries became positive during the 1990s. Although these countries’ relative growth in patent quality faded away in the 2000s, notice that the corresponding coefficient values are still similar to or better than those of Japan and the advanced European nations.

For China, a similar surge was observed during the 2000s. On adjusted log average citations, China's patent quality gap against the US became statistically insignificant in this decade. Our findings are consistent with evidence reported by Griffith and Miller (2011), who showed that the proportion of EPO patent applications with at least one Chinese inventor that are near science – more fundamental research and hence, presumably higher quality – was higher than that of all EPO patent applications for the period of 1995–2005.^20^

Finding 2 Patent quality of the advanced economies of Europe and Japan declined relative to the US.

The second notable finding is the relative decline of the advanced nations of Europe and Japan. In the initial sample decade of the 1980s, EU and the major European countries (Germany, France, UK and Switzerland) had relatively small patent quality deficits against the US in terms of log average citations. For instance, the coefficient value was −0.150 for Germany and −0.151 for UK, implying that the average citations of these two countries were about 14% lower than that of the US. Japanese patent quality in this measure was actually better, by about 18%, than the US. However, a significant decline occurred during the 1990s for all these countries and this downward trend continued in the 2000s. For Germany, the coefficient dropped to −0.363(≈ −30%) in the 1990s and −0.529(≈ −41%) in the 2000s, and for UK, it became −0.304(≈ −26%) and −0.344(≈ −29%) respectively, with all these estimates being significant at 0.1% level. For Japan, the initial advantage was erased statistically by the 1990s and moved into deficit in the subsequent decade.

The same downward trend is evident also in terms of adjusted log average citations. Here, this group of advanced countries all began with superior patent quality than the US but steady decline led to only one of them (UK) with a positive coefficient during the latest decade.

The remaining countries did not reveal patterns that were as striking or conclusive as the aforementioned countries. Canada and Australia showed some marginal gains relative to the US but the changes were either small or not showing consistent trends across the two measures of patent quality. Israel, India and Former Soviet Union states as well as the Rest of the World did record consistent and steady improvements in their patent quality. While this observation reinforces our first finding and paints a broad upward pattern for the emerging economies as a whole, the gains achieved by these nations fall somewhat short of the kind of rapid quality surges experienced by the patents from Korea, Taiwan and China.

Finding 3 Patent quality of the US has strengthened in terms of its relative position against the world.

Finally, what can we learn from the results about US patents? If one considers average citations from all patents (home or otherwise), the US patent quality was higher than every country except Japan in the 1980s and this dominance was maintained throughout. In particular, it is worthwhile to notice that, despite the gains made by certain economies, there is no single country whose corresponding coefficient is better than −0.218 in the 2000s (there were four such countries in the 1980s). In the final decade, the coefficient estimates range between −0.890 and −0.218 while in the 1980s, the corresponding range is substantially more dispersed and spans −2.088 to 0.203.

With adjustment of home bias, the levels of country coefficients indeed shift up but the distribution of the coefficients demonstrates similar trends. The range shrinks from [−1.663, 0.397] in the 1980s to [−0.490, 0.287] in the 2000s for the regression on log adjusted average citations. The number of countries with statistical patent quality surplus against the US is just three in the 2000s (not counting the rest of the world), down from seven in the 1980s.

Overall, our results suggest strengthening of the US position in the global patent quality ladder since 1980. Most of the countries that made inroads against the US are those who were initially far behind and hence jumped onto the process of catching up. The traditional powerhouses in innovation, Japan and the major European countries, have continued to decline against the US. It is also illuminating to observe that the rapid catching‐up by Korea and Taiwan in the 1990s actually reversed in the 2000s.

The main regression results here are intact if we control for aggregate sectors (six industrial categories as defined by the NBER data) instead of using sub‐category sectors and also if we use a five‐year window to compute both measures of the average citations instead of using the two‐year window. See Tables C1 and C2 in online Appendix C.

### 2.2 Hirsch Index

While average citations capture some aspect of a country's patent quality, such a measure may not explain overall technological innovation because they fail to take into account the total size or productivity of the innovation sector, i.e. the number of patents granted. One measure to capture citation‐adjusted total research output is the Hirsch index, or simply H‐index, which is widely used to measure a scholar's research performance.^21^

In online Appendix B, we report two sets of estimation results (Poisson and negative binomial models) with H‐indices as dependent variables. These results broadly support our previous findings on average patent quality. With the quantity of patents also taken into account, we see that Korea and Taiwan actually continued to strengthen their worldwide positions during the 2000s; moreover, substantial technological gaps still separate the emerging economies from the advanced economies of Japan and Europe. In order to highlight these overall trends, in Table 3, we present the international rankings based on H‐index regression results for each of the three previous decades. A higher ranking means an H‐index closer to that of the US in the corresponding decade. We observe that Japan, Germany and EU maintained the top three positions throughout the three decades. The rankings of Korea and Taiwan rose most substantially, while France and Switzerland showed notable declines.

**Table 3:** Hirsch Index Rankings

	Hirsch‐index rankings		
Country	1980–9	1990–9	2000–9
Japan (JP)	1	1	1
Germany (DE)	2	2	2
EU	3	3	3
France (FR)	5	6	8
United Kingdom (UK)	4	4	5
Taiwan (TW)	10	7	6
Korea (KR)	13	9	7
Canada (CA)	6	5	4
Switzerland (CH)	7	8	12
Australia (AU)	9	12	11
Israel (IL)	11	11	10
China (CN)	14	14	13
India (IN)	15	15	15
Former Soviet Union (FSU)	12	13	14
Rest of the World (RW)	8	10	9

*Notes*. These ranks are based on our regression model with the Hirsch index of each location as the dependent variable. We rank each country in descending order according to its coefficient estimate for each decade.

## 3 Citation Lags

The patent citations data enable us to explore another channel of measuring the trends in technological progress. Taking US as the knowledge frontier, we next consider the speed with which US patents are cited by non‐US patents.

### 3.1 Econometric Model

We adopt the fixed‐effects estimator of Griffith *et al*. (2011). There are a set of inventions *i* = 1, …, *I* and a set of inventors *j* = 1,…, *J*. The inventors will learn of invention *i* after a time period $T_{ij}$ and therefore, $T_{ij}$ can be thought of as the ‘citation (or diffusion) lag’ between invention *i* and inventor *j*. There are several factors which determine the citation lag including characteristics of the invention *i*, $Z_{i}$, characteristics of the inventor *j*, $Z_{j}$ and the joint characteristics of the invention‐inventor match, $Z_{ij}$. There will be a set of non‐geographical variables as well as geographical variables that will influence the speed at which technology spillover occurs.

The hazard function of the citation lag is affected by a vector of explanatory variables $X_{ij}$, incorporating the empirically observable counterparts to $Z_{ij}$ and $Z_{j}$ and an unobservable fixed effect, $U_{i}$, which absorbs all the factors specific to the cited patent, $Z_{i}$. Unobserved heterogeneity $U_{i}$ includes patent quality among other things.

We take the set of US patents as the frontier and regress US (cited) patents on characteristics of citing patents from non‐US inventors. Since higher quality patents are likely to be cited more quickly, it is crucial to control for unobserved patent quality, as emphasised by Griffith *et al*. (2011).^22^ As a consequence, our estimates would be robust even if there had been changing trends in the quality of US patents over time.

Specifically, Griffith *et al*. (2011) consider a multiple‐spell version of the mixed proportional hazards model. Their regression model can be written as

(2) $$\log\Lambda_{i}\left(T_{ij}\right)=-X_{ij}^{\prime }\beta -U_{i}+\varepsilon_{ij},$$

where ***β*** is a vector of unknown parameters, $\lambda_{i}\left(\cdot \right)$ is a cited‐patent specific baseline hazard function, $\Lambda_{i}\left(t\right)\;=\;\int_{0}^{t}\lambda_{i}\left(u\right)du,\varepsilon_{ij}$ is i.i.d. over *i* and *j*, independent of $X_{ij}$, and $\varepsilon_{ij}$ has the cumulative distribution function $F\left(\varepsilon_{ij}\right)\;=\;1-\exp\left[-\exp\left(\varepsilon_{ij}\right)\right]$. Then an estimate of ***β*** can be obtained using a conditional likelihood approach, while accounting for right censoring.

### 3.2 Regression Results

In our empirical analysis, we fix the ‘potentially cited’ country to be the US and consider only US patents. As discussed in the previous subsection, this amounts to interpreting the US as the knowledge frontier. This simplifying assumption is a reasonable first‐order approximation in view of our findings in Section 2. We split the sample period into eight sub‐periods of each lasting four years and estimate for each sub‐period the citation lag model with the first two citations (*J* = 2), as described in the previous subsection.

Included covariates for citing patents are the self citation indicator (whether a citation is from the identical assignee), the same sub‐category dummy (whether a citation is from the same sub‐category), the base cohort size (which is the number of patents in the citing country and technology sub‐category for the citing year), the corporation dummy (whether the citing first assignee is a corporation or not), category dummies (six industry level dummies) and citing country dummies. Among these, the citing country dummies are covariates of interest. Their estimated coefficients represent how fast the non‐US inventors cite US patents compared to US inventors and may also indicate how close the non‐US inventors are to the technology frontier.

Table 4 shows the estimation results.^23^ For example, the first row of Table 4 displays the coefficient estimates for Japan through the eight sub‐periods. The estimate for the first sub‐period in this row is −0.21 which means that inventors in Japan cited US patents about 21% slower than US inventors.

**Table 4:** Main Estimation Results of the Citation Lag Model

	Period							
	1	2	3	4	5	6	7	8
Country	(1980–3)	(1984–7)	(1988–91)	(1992–5)	(1996–9)	(2000–3)	(2004–7)	(2008–11)
Japan (JP)	−0.21	−0.13	−0.12	−0.23	0.03	0.03	−0.11	−0.01
	(0.02)	(0.02)	(0.02)	(0.02)	(0.02)	(0.02)	(0.03)	(0.06)
Germany (DE)	−0.29	−0.25	−0.11	−0.24	−0.01	0	−0.12	−0.28
	(0.03)	(0.03)	(0.03)	(0.03)	(0.03)	(0.03)	(0.04)	(0.1)
EU	−0.26	−0.2	−0.2	−0.31	−0.05	−0.05	−0.22	−0.13
	(0.03)	(0.03)	(0.03)	(0.03)	(0.03)	(0.03)	(0.04)	(0.1)
France (FR)	−0.22	−0.08	0.01	−0.03	0.13	0.11	0.06	0.07
	(0.02)	(0.02)	(0.01)	(0.01)	(0.01)	(0.01)	(0.02)	(0.05)
United Kingdom (UK)	−0.41	−0.3	−0.26	−0.32	−0.03	−0.08	−0.15	−0.19
	(0.03)	(0.03)	(0.02)	(0.02)	(0.02)	(0.02)	(0.03)	(0.09)
Taiwan (TW)	−1.23	−1.04	−0.5	−0.35	0.09	−0.03	−0.11	−0.02
	(0.11)	(0.09)	(0.06)	(0.04)	(0.03)	(0.03)	(0.03)	(0.07)
Korea (KR)	−0.9	−0.67	−0.33	−0.22	0.13	0.19	0.06	0.09
	(0.08)	(0.05)	(0.04)	(0.03)	(0.02)	(0.02)	(0.03)	(0.08)
Canada (CA)	−0.48	−0.33	−0.69	−0.9	−0.5	−0.48	−0.52	−0.11
	(0.06)	(0.06)	(0.16)	(0.14)	(0.11)	(0.07)	(0.06)	(0.1)
Switzerland (CH)	−0.44	−0.37	−0.37	−0.43	−0.1	−0.28	−0.4	−0.18
	(0.03)	(0.03)	(0.06)	(0.06)	(0.05)	(0.05)	(0.09)	(0.2)
Australia (AU)	−0.22	−0.17	−0.34	−0.36	−0.08	−0.11	−0.35	−0.22
	(0.04)	(0.05)	(0.03)	(0.02)	(0.02)	(0.02)	(0.03)	(0.07)
Israel (IL)	−0.7	−0.61	−0.12	−0.31	0.07	−0.08	−0.13	0.27
	(0.09)	(0.07)	(0.05)	(0.05)	(0.05)	(0.05)	(0.07)	(0.16)
China (CN)			−0.37	−0.41	−0.24	−0.17	−0.44	−0.31
			(0.06)	(0.05)	(0.04)	(0.04)	(0.05)	(0.11)
India (IN)			−0.79	−0.63	−0.28	−0.4	−0.44	−0.11
			(0.21)	(0.17)	(0.11)	(0.08)	(0.09)	(0.12)
Former Soviet Union (FSU)	−0.13	−0.64	−0.34	−0.33	−0.09	−0.22	−0.38	−0.19
	(0.12)	(0.14)	(0.13)	(0.11)	(0.1)	(0.09)	(0.13)	(0.31)
Rest of the World (RW)	−0.57	−0.51	−0.44	−0.54	−0.2	−0.15	−0.33	−0.22
	(0.05)	(0.05)	(0.05)	(0.04)	(0.04)	(0.03)	(0.04)	(0.1)

*Notes*. The reported numbers are the estimated coefficient values for the country dummy estimated from the regression results above. In the parentheses are standard errors. Our regression model controls for base cohort size (the number of patents in the citing country and technology sub‐category for the citing year), self citations (citations between patents with identical assignees) and within‐sub‐category citations (citations between patents within the same category). Corporation dummies (whether or not the first assignee is a corporation) and category dummies are also included. We include CN and IN in RW for the first two periods to avoid diverging estimators due to the small sample sizes of such cohorts.

**Figure 2:**
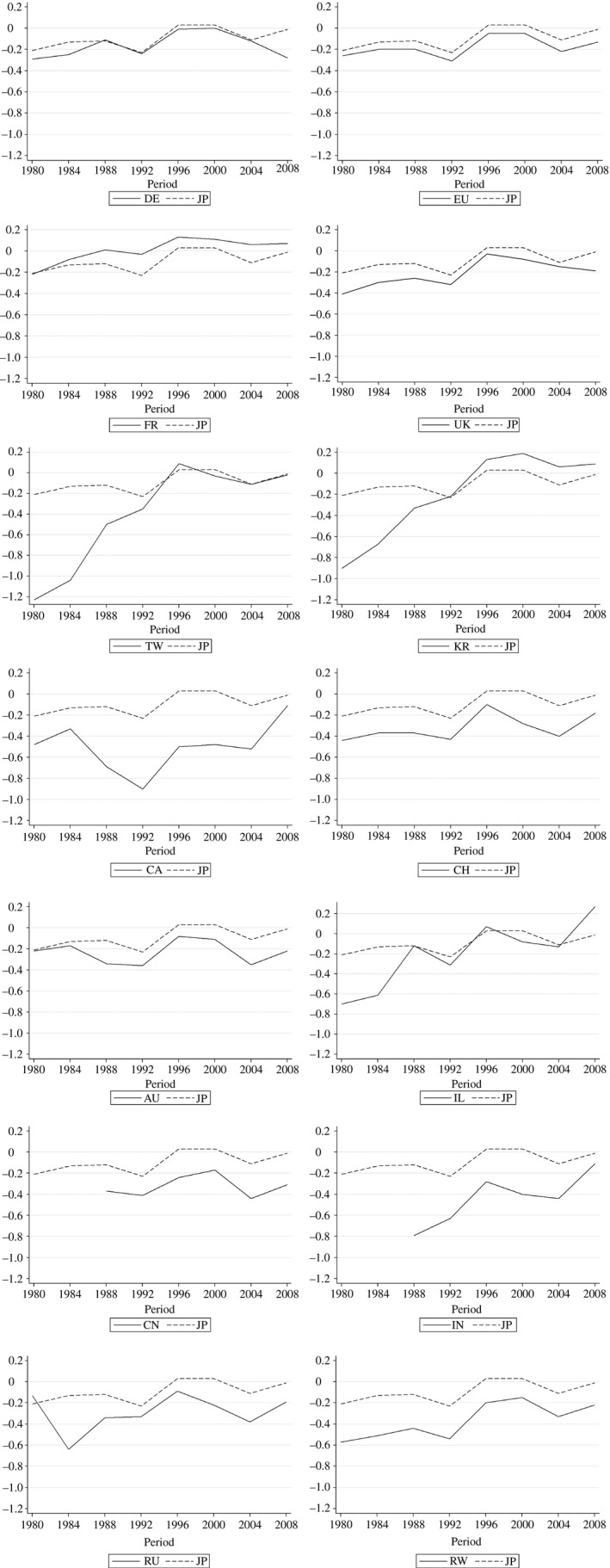
Graphical Representation of Estimation Results in Table 4 *Notes*. Each graph plots the coefficient values for the country dummy. Japan is included in every graph as a benchmark.

To assist exposition, Figure 2 displays the coefficients of each country dummy together with the corresponding coefficients for the Japanese dummy. In terms of citation lags, Japan is one of the countries that has maintained its proximity to the US throughout the sample period.

We summarise our main finding for this Section below:

Finding 4 Most significant gains in narrowing of the citation lag against the knowledge frontier were made by the emerging economies of Israel, Korea and Taiwan.

Korea, Taiwan and Israel all began the sample period with citation lags substantially below that of Japan; for instance, during 1980–3, Taiwanese inventors cited US patents about 120% slower than US inventors, which amounted to 100% lag relative to Japan. By 1996–9, however, all three countries caught up with Japan, and in the case of Korea and Israel, the deficits reversed during the 2000s. Although improved communication technology may have led to general decline in citation lag (i.e. ‘death of distance’ as identified in Griffith *et al*. (2011)), the asymmetric progress made by this league of countries points to real closing of the citation lag against the knowledge frontier. Note that China does not feature in this league. Although China closed the citation lag somewhat during the 1990s, the distance grew again in the last decade.

It is also interesting to observe that, in terms of citation speed, the advanced economies of Japan and Europe (as well as Canada, Australia and FSU) actually maintained their relatively close position to the US frontier throughout the sample periods. Japan, in particular, have been more or less on level terms with the US for the last three decades, ahead of all the other countries. Canadian inventors are slower than Japanese inventors, despite their geographic proximity to the US.

Our findings are related to the literature on ‘absorptive capacity’. Griffith *et al*. (2003, 2004) found evidence that R&D is statistically and economically important in enhancing technology transfer as well as stimulating innovation directly. Mancusi (2008) investigated the issue of international knowledge spillovers and absorptive capacity. Using EPO patent applications and their citations from 1978 to 2003, Mancusi (2008) concluded that absorptive capacity increases the elasticity of a catching‐up country's innovation to international spillovers but not that of an advanced country. Our estimation results are consistent with this perspective. In particular, unlike Korea, Taiwan and Israel, other emerging countries such as China might not have accumulated a sufficient level of absorptive capacity to benefit from international knowledge spillovers.

One might suspect that our results might be driven by a small number of industry sectors since the economies of Korea and Taiwan, in particular, are highly concentrated (e.g. electronics and computers). In order to address this issue, we also conduct the citation lag model regression sector‐wise and report its results in online Appendix C (Tables C3–C8). The magnitudes of the coefficients of interest turn out to differ across industries; for instance, the coefficients for Taiwan and Korea in the first sub‐period are −1.12 and −0.48, respectively, for the chemical sector, while the corresponding numbers for the computers and communication sector are −2.65 and −2.2 respectively. However, in terms of the trends, these sector‐wise regression results are qualitatively similar to those of Table 4. In particular, we observe rapid upward trends for Korea, Taiwan and Israel in all six sectors.^24^

## 4 Conclusion

In this article, we analyse cross‐country trends in some aspects of technological progress over the period of 1980–2011 by considering an updated USPTO citations dataset. Our estimation results reveal several noteworthy stylised facts. As widely expected, the emerging Asian economies of Korea, Taiwan and China have indeed achieved rapid inroads towards the technology frontier. In the case of Korea and Taiwan, progress has been made in terms of patent quality as well as citation lag. Chinese patents are on average of substantially higher quality now than before but Chinese inventors have yet to reduce the citation lag relative to the frontier. In contrast, advanced economies of Europe and Japan have been in steady decline in their patent quality. Finally, the US has strengthened its position in the international patent quality ladder.

Our results pose a number of intriguing questions for the years ahead. On the one hand, the emerging Asian countries would overtake the traditional knowledge powerhouses of Europe and Japan if they could sustain the recent levels of faster technological progress. On the other hand, it will be interesting to see whether these emerging countries do close the gap further against the US. Although Korea and Taiwan managed to elevate the quality of their patents to the US level in the 1990s, the US actually pushed ahead in the 2000s. The Korean and Taiwanese experience was repeated by China in the 2000s. Would the US demonstrate another bout of technological resilience in the current decade against China? Last, but not least, we also wait to see if other countries emerge to join the growth path of the aforementioned Asian economies in innovation.

In this study, we focus on patent citations data from the USPTO. While this may cause some bias in favour of US patents, the trends of all other countries are measured relative to those of the US. We also consider adjusted average citation rates to account for the home bias. Nonetheless, it would be worthwhile also to study the corresponding trends using data from other major patent offices, especially, from the EU.

Another extension would be to consider other measures of patent quality beyond citation rates, such as market value. Although citations are correlated with the market value of a patent, the latter indicator is affected in important ways by other variables. For example, Hall and Ziedonis (2001) found that, in certain sectors, individual patents only have value if they are part of a company's broader patent portfolio. More generally, one might also like to estimate the trends in overall technological progress beyond those captured by patents. Not all industries rely equally on the patent system in appropriating their R&D investments.

Perhaps more important future research prompted by the current study would be to investigate the sources of technological progress that can help explain the reported cross‐country variations. Meaningful insights could be obtained by linking the patent citations data with data on potentially important contributors of knowledge growth, such as R&D and higher education expenditures.

## Supporting information


**Appendix A.** Data.
**Appendix B.** Hirsch Index for Patent Citations.
**Appendix C.** Robustness Check.

**Data S1.**

## Notes

1 Table 1 presents the total number of utility patents granted by USPTO to each country during 1980–2011. Despite the apparent global success in patent production, serious concerns have been raised against its actual substance. See, for instance, Federal Trade Commission (2003), Jaffe and Lerner (2004) and Merrill *et al*. (2004). For quality decline in patents from the European Patent Office (EPO), see Eaton *et al*. (2004), Guellec and de La Potterie (2007), Jones (2009), Griffith and Miller (2011) and OECD (2011).

2 Several studies have also established a statistical correlation between citation rates and market value of a patent (Bloom and Van Reenen, 2002; Hall *et al*., 2005; Gambardella *et al*., 2008; Belenzon, 2012).

3 Another potential drawback of using patent data is that the grant date does not accurately reflect when the invention took place. Our use of 10‐year windows partially addresses this issue.

4 Other countries that also demonstrate patent quality improvement, albeit at less striking patterns than Korea, Taiwan and China, include Israel and India.

5 See Griliches (1990), Jaffe and Trajtenberg (2002) and Hall and Harhoff (2012), among others.

6 For another early application of citation data, see Lieberman (1987).

7 These datasets have been widely used in the economic analysis of knowledge spillovers. See Hall *et al*. (2001) for an overview.

8 Hall's augmented datasets – specifically, PAT63_02 and CITE75_02 – comprise of all 3,416,957 utility patents granted by the USPTO between January 1963 and December 2002 by the USPTO and all 22,309,440 citations made by utility patents granted in the period of January 1975 to December 2002. Hall's datasets are available at http://elsa.berkeley.edu/b~hhall/patents.html.

9 Roughly 99% of all patents in the sample period originated from the 15 countries. Before 1980, there were only very few patents from the emerging economies of China, India, Korea, Taiwan and others.

10 EU consists of the following 12 countries: Austria, Belgium, Denmark, Finland, Greece, Ireland, Italy, Luxembourg, Netherlands, Portugal, Spain and Sweden, as in Griffith *et al*. (2011).

11 Hong Kong is excluded from China. In online Appendix A, we explain how the location of the first inventor of a Chinese patent was checked in this regard.

12 For consistency throughout the periods, we regard all patents whose inventors were located in the 15 former Soviet Union member countries as ‘FSU (Former Soviet Union)’ patents.

13 There are all 16 countries (or groups of countries) including RW (Rest of the World), 37 sectors (sub‐categories) and 30 grant years (excluding the last couple of years so that patents for each grant year have a two‐year‐period of citations received).

14 Sub‐category is a variable from the NBER datasets and it sub‐categorises the six industrial categories. There are 37 sub‐categories in total.

15 While the dataset covers all 32 years from 1980 to 2011, our regression here considers patents up to only 2009 to allow for full two‐year forward citations; also, year 1980 is omitted.

16 We have also conducted DiD estimation across different time intervals (periods of 8, 9, 11 and 12 years) but the results are qualitatively unaffected.

17 For cells with no average citations, we replace the zeros with a small positive number and run weighted regressions. Our results are not sensitive, both qualitatively and quantitatively, on the choice of this parameter. Using left‐censored tobit regressions instead of OLS also does not affect the results.

18 The size of the coefficient matrix is (15 × 1) instead of (16 × 1) since we omit the US benchmark case.

19 Since we consider the logarithm of average citations in columns (1)–(3) in Table 2, we can translate the coefficients as percentage differences relative to the US by [exp(coefficient) − 1] × 100.

20 Griffith and Miller (2011) attributed part of the success of China to investment of foreign multinationals. See also Hu and Jefferson (2009) for explanations behind China's patent surge.

21 An author with a total of *n* papers has H‐index *h* if he or she has more than *h* papers that have been cited at least *h* times and the other *n* − *h* papers have been cited at most *h* times. This measure is originally proposed by Hirsch (2005) to analyse individuals’ research output in physics. In economics, Ellison (2013) recently used Hirsch‐like indices to examine how well different indices explain labour market outcomes for young, tenured economists at 50 US departments.

22 To this end, we only use ‘ within‐cited‐patent’ variations.

23 We include China and India as part of the Rest of the World for the first two periods to avoid imprecisely estimated estimators due to the small sample sizes of such cohorts.

24 In online Appendix C, we also report estimation results of the citation lag model based on the second and third citations (Table C9) as well as on the third and fourth citations (Table C10). Our main qualitative findings are mostly unaffected.
