# Supplementary material for: International Trends in Technological Progress: Evidence from Patent Citations, 1980–2011
Source: Econ J (London). 2017 Oct 24;127(605):F50–70. doi: 10.1111/ecoj.12314 (PMC5699438; doi:10.1111/ecoj.12314)
Supplement: Supplementary file 1 — Appendix A. Data. Appendix B. Hirsch Index for Patent Citations. Appendix C. Robustness Check. [file ECOJ-127-F50-s001.pdf]

The Economic Journal, Doi: 10.1111/ecoj.12314 © 2017 The Authors. The Economic Journal published by John Wiley & Sons Ltd on behalf of Royal Economic Society. Published by John Wiley & Sons, 9600 Garsington Road, Oxford OX4 2DQ, UK and 350 Main Street, Malden, MA 02148, USA.

Technical Appendix to

INTERNATIONAL TRENDS IN TECHNOLOGICAL PROGRESS: EVIDENCE FROM PATENT CITATIONS, 1980–2011

Soonwoo Kwon, Jihong Lee and Sokbae Lee

Economic Journal, doi: 10.1111/ecoj.12314

These online Appendices offer further details on the dataset and report some additional analyses that are left out of the main text for expositional reasons. Some equations and Tables that we refer to below appear in the main text.

Appendix A. Data

We extended the NBER data file, PAT63_99, to include 12 additional years up to 31 December 2011.[1] Our data file is named PAT63_11, and it mainly builds on Brownyn Hall's data file, PAT63_02. While the NBER Patent Data Project provides a dataset that includes patents granted up to 2006 (PAT76_06), this does not contain the inventor location of each patent (https://sites.google.com/site/patentdataproject/). Likewise, we also extended CITE75_99, the pairwise citations data, to CITE75_11. Our data files are constructed from Google's USPTO Bulk Download service that makes publicly available in XML format bibliographic data of all patents granted since 1976 to present (https://www.google.com/googlebooks/uspto.html). We used Python to parse the XML files and extracted the necessary data for our analysis. Tag names for pre-2006 patents that are in codes were deciphered for accurate extraction using USPTO's codebook.

We extracted the following information for each patent: patent number, grant date, three-digit main classification, assignee, assignee code, inventor country and patents that the given patent cited. See Hall *et al.* (2001) for details of each variable. Using such raw data, we constructed 'subcat' variables, following Hall *et al.* (2001). The three-digit classification code was matched with each subcat code as provided in the NBER webpage. There were also some new classification codes in the recent years that do not have corresponding subcat codes. For these, we used the match applied in PAT76_06 when possible, and for those that had no matches we just left the subcat variable as missing. The number of patents with missing subcat, which appear only after 2000, does not exceed 80 for each year.

The patent counts did not match with the USPTO statistics for China and Hong Kong. This was mainly due to inconsistent reports of the patent claimers. For example, while the USPTO maintains separate accounts for China and Hong Kong, some Hong Kong inventors reported their country as China. We identified the same problem in PAT63_02 for patents granted later than 2000. Also, there were some inventors from Taiwan and Singapore who reported their location as China. In order to rectify this problem, we extracted an extra

[1] The last grant date in the dataset is 27 December 2011 since USPTO grants patents on a weekly basis.

variable, inventor city and manually cleaned up the discrepancies. After this clean-up process, the patent counts by country and grant year became consistent with the USPTO statistics.

Appendix B. Hirsch Index for Patent Citations

Table B1 reports the H-index of each country for the eight sub-periods within 1980–2011. The US strictly dominates all other countries in all periods; the growing trends of the developing countries such as China, India, Korea and Taiwan are also evident.

We run Poisson regression and negative binomial regression with the H-indices as the dependent variable to examine the trends observed in Table B1. We use the following model specification

$$Y_{cst} = \exp\left(\alpha_0 + \Sigma_{i=1}^{36}\alpha_i s_i + \Sigma_{i=1}^{29}\beta_i t_i + \boldsymbol{\eta}_1' \boldsymbol{X}_{cst,80} + \boldsymbol{\eta}_2' \boldsymbol{X}_{cst,90} + \boldsymbol{\eta}_3' \boldsymbol{X}_{cst,00}\right) + \varepsilon_{cst},$$

where the variables on the right-hand side are defined as in (1).[2]

Here, the dependent variable, Y_{cst}, is the H-index for a given country c, sector s and year t. For example, if $y_{\mathrm{JP},11,1980} = h$, this means that considering the patents granted in 1980 for

Table B1
Hirsch Index

Country	Period 1 (1980–3)	2 (1984–7)	3 (1988–91)	4 (1992–5)	5 (1996–9)	6 (2000–3)	7 (2004–7)	8 (2008–11)
United States (US)	193	233	277	317	336	268	150	56
Japan (JP)	99	117	137	148	168	126	72	26
Germany (DE)	76	88	95	106	99	88	54	20
EU	73	88	90	102	118	103	60	22
France (FR)	70	74	89	95	95	79	44	18
United Kingdom (UK)	70	81	101	103	108	94	60	19
Taiwan (TW)	19	33	47	64	76	77	52	15
Korea (KR)	12	21	35	63	75	78	51	21
Canada (CA)	62	77	87	108	111	96	52	19
Switzerland (CH)	58	58	62	71	67	51	33	11
Australia (AU)	36	42	49	65	60	69	38	24
Israel (IL)	34	39	53	73	87	89	51	20
China (CN)	5	11	27	20	21	24	26	14
India (IN)	8	10	11	19	22	27	25	10
Former Soviet Union (FSU)	30	32	28	33	39	33	20	7
Rest of the World (RW)	45	51	53	64	81	70	44	15

Notes. The sample period is divided into eight equal-length sub-periods and we consider each country of each sub-period as an ‘author’ to calculate the H-index for each ‘author’. An author with a total of n patents has H-index h if he has more than h patents that have been cited at least h times and the other $n - h$ patents have been cited at most h times.

[2] As in (1), there are 36 sub-category dummies and 29 year dummies.

© 2017 The Authors.
The Economic Journal published by John Wiley & Sons Ltd on behalf of Royal Economic Society.

subsection category 11 by Japanese inventors as patents of one big inventor, the H-index is *h*. Since this dependent variable is count data, we apply Poisson regression and negative binomial regression to estimate the coefficients. The standard errors are again clustered by country and sub-category.

The results are shown in Table B2. These results are broadly consistent with our findings on citation frequency and average citations. The main regression results remain unaffected if we control for aggregate sectors instead of using sub-category sectors. See Table B3.

Table B2

Regression Results for Hirsch Index

	Method					
	Poisson regression			Negative binomial regression		
Country	(1) 1980–9	(2) 1990–9	(3) 2000–9	(4) 1980–9	(5) 1990–9	(6) 2000–9
Japan (JP)	−0.596***	−0.673***	−0.763***	−0.601***	−0.670***	−0.752***
	(0.040)	(0.054)	(0.078)	(0.040)	(0.057)	(0.082)
Germany (DE)	−0.952***	−1.214***	−1.234***	−0.947***	−1.198***	−1.214***
	(0.050)	(0.052)	(0.059)	(0.048)	(0.052)	(0.063)
EU	−1.158***	−1.321***	−1.256***	−1.158***	−1.319***	−1.246***
	(0.049)	(0.048)	(0.063)	(0.046)	(0.046)	(0.065)
France (FR)	−1.304***	−1.488***	−1.591***	−1.308***	−1.478***	−1.573***
	(0.043)	(0.041)	(0.062)	(0.042)	(0.041)	(0.067)
United Kingdom (UK)	−1.210***	−1.433***	−1.402***	−1.215***	−1.431***	−1.389***
	(0.045)	(0.047)	(0.066)	(0.043)	(0.048)	(0.070)
Taiwan (TW)	−2.506***	−1.827***	−1.528***	−2.519***	−1.820***	−1.505***
	(0.108)	(0.091)	(0.105)	(0.105)	(0.090)	(0.104)
Korea (KR)	−3.167***	−2.047***	−1.556***	−3.186***	−2.054***	−1.538***
	(0.071)	(0.097)	(0.101)	(0.071)	(0.095)	(0.103)
Canada (CA)	−1.479***	−1.468***	−1.352***	−1.486***	−1.465***	−1.341***
	(0.064)	(0.052)	(0.070)	(0.061)	(0.050)	(0.071)
Switzerland (CH)	−1.677***	−1.973***	−2.023***	−1.676***	−1.960***	−2.004***
	(0.074)	(0.070)	(0.067)	(0.072)	(0.069)	(0.070)
Australia (AU)	−2.259***	−2.305***	−1.970***	−2.269***	−2.303***	−1.945***
	(0.087)	(0.072)	(0.097)	(0.083)	(0.071)	(0.104)
Israel (IL)	−2.525***	−2.278***	−1.831***	−2.545***	−2.294***	−1.831***
	(0.067)	(0.076)	(0.108)	(0.065)	(0.074)	(0.109)
China (CN)	−3.530***	−3.633***	−2.609***	−3.546***	−3.631***	−2.575***
	(0.075)	(0.084)	(0.102)	(0.072)	(0.080)	(0.104)
India (IN)	−3.675***	−3.671***	−2.855***	−3.694***	−3.665***	−2.823***
	(0.082)	(0.126)	(0.122)	(0.083)	(0.124)	(0.125)
Former Soviet Union (FSU)	−2.722***	−3.116***	−2.828***	−2.731***	−3.111***	−2.804***
	(0.078)	(0.068)	(0.077)	(0.076)	(0.067)	(0.080)
Rest of the World (RW)	−1.986***	−2.075***	−1.752***	−1.992***	−2.069***	−1.731***
	(0.070)	(0.051)	(0.070)	(0.066)	(0.051)	(0.074)

Notes. Columns (1)–(3) show estimated coefficient values for the three DiD regressors from Poisson regression, and columns (4)–(6) contain corresponding estimates from negative binomial regression. $^{*}p < 0.05$, $^{**}p < 0.01$, $^{***}p < 0.001$. In the parentheses are standard errors. Grant year dummies and sub-category dummies are also included as regressors. We use standard errors clustered by country and sub-category. The main results are not sensitive to the choice of standard errors. For robustness we conduct both Poisson regression and negative binomial regression, which are standard methods in count data analysis.

© 2017 The Authors.
The Economic Journal published by John Wiley & Sons Ltd on behalf of Royal Economic Society.

Table B3

Regression Results for Hirsch Index (with category dummies)

	Method					
	Poisson regression			Negative binomial regression		
Country	(1) 1980–9	(2) 1990–9	(3) 2000–9	(4) 1980–9	(5) 1990–9	(6) 2000–9
Japan (JP)	−0.596***	−0.673***	−0.763***	−0.603***	−0.669***	−0.749***
	(0.068)	(0.083)	(0.095)	(0.069)	(0.084)	(0.095)
Germany (DE)	−0.952***	−1.214***	−1.234***	−0.936***	−1.186***	−1.194***
	(0.077)	(0.085)	(0.087)	(0.074)	(0.082)	(0.086)
EU	−1.158***	−1.321***	−1.256***	−1.144***	−1.305***	−1.224***
	(0.078)	(0.087)	(0.096)	(0.077)	(0.085)	(0.094)
France (FR)	−1.304***	−1.488***	−1.591***	−1.296***	−1.467***	−1.557***
	(0.075)	(0.080)	(0.087)	(0.073)	(0.077)	(0.086)
United Kingdom (UK)	−1.210***	−1.433***	−1.402***	−1.201***	−1.420***	−1.372***
	(0.078)	(0.085)	(0.093)	(0.077)	(0.083)	(0.092)
Taiwan (TW)	−2.452***	−1.824***	−1.525***	−2.453***	−1.801***	−1.477***
	(0.119)	(0.115)	(0.122)	(0.119)	(0.113)	(0.120)
Canada (CA)	−1.477***	−1.468***	−1.352***	−1.462***	−1.447***	−1.324***
	(0.095)	(0.091)	(0.096)	(0.092)	(0.088)	(0.094)
Korea (KR)	−3.113***	−2.043***	−1.556***	−3.119***	−2.047***	−1.517***
	(0.090)	(0.119)	(0.119)	(0.088)	(0.117)	(0.119)
Switzerland (CH)	−1.675***	−1.971***	−2.019***	−1.657***	−1.940***	−1.973***
	(0.095)	(0.097)	(0.099)	(0.093)	(0.095)	(0.098)
Australia (AU)	−2.248***	−2.298***	−1.967***	−2.233***	−2.277***	−1.912***
	(0.113)	(0.107)	(0.119)	(0.112)	(0.107)	(0.120)
Israel (IL)	−2.506***	−2.271***	−1.825***	−2.508***	−2.273***	−1.808***
	(0.107)	(0.118)	(0.135)	(0.107)	(0.115)	(0.133)
China (CN)	−3.431***	−3.556***	−2.591***	−3.432***	−3.536***	−2.510***
	(0.108)	(0.118)	(0.123)	(0.108)	(0.119)	(0.127)
India (IN)	−3.585***	−3.591***	−2.821***	−3.583***	−3.568***	−2.754***
	(0.082)	(0.137)	(0.136)	(0.077)	(0.134)	(0.138)
Former Soviet Union (FSU)	−2.700***	−3.083***	−2.798***	−2.688***	−3.064***	−2.744***
	(0.106)	(0.101)	(0.107)	(0.104)	(0.101)	(0.108)
Rest of the World (RW)	−1.987***	−2.074***	−1.752***	−1.970***	−2.050***	−1.703***
	(0.099)	(0.090)	(0.100)	(0.095)	(0.088)	(0.099)

Notes. *p < 0.05, **p < 0.01, ***p < 0.001. The specifications are the same as for Table B2 except that here we include category dummies instead of sub-category dummies.

© 2017 The Authors.
The Economic Journal published by John Wiley & Sons Ltd on behalf of Royal Economic Society.

Appendix C. Robustness Check

Table C1

Regression Results (log average citations, with category dummies)

	Dependent variable					
	Log average citations			Log average citations (adjusted)		
Country	(1) 1980–9	(2) 1990–9	(3) 2000–9	(4) 1980–9	(5) 1990–9	(6) 2000–9
Japan (JP)	0.214**	−0.064	−0.251***	0.367***	0.146**	−0.072
	(0.076)	(0.075)	(0.064)	(0.060)	(0.055)	(0.052)
Germany (DE)	−0.175*	−0.402***	−0.558***	0.236***	0.050	−0.135*
	(0.075)	(0.073)	(0.062)	(0.069)	(0.066)	(0.056)
EU	−0.294***	−0.423***	−0.498***	0.241***	0.112	−0.019
	(0.066)	(0.074)	(0.075)	(0.060)	(0.063)	(0.081)
France (FR)	−0.312***	−0.472***	−0.652***	0.250***	0.056	−0.161*
	(0.065)	(0.072)	(0.068)	(0.060)	(0.064)	(0.071)
United Kingdom (UK)	−0.173*	−0.341***	−0.375***	0.380***	0.247***	0.168*
	(0.078)	(0.072)	(0.076)	(0.066)	(0.062)	(0.083)
Taiwan (TW)	−0.596***	0.031	−0.237**	−0.214**	0.331***	0.025
	(0.082)	(0.111)	(0.090)	(0.077)	(0.077)	(0.074)
Korea (KR)	−1.372***	−0.250*	−0.350***	−0.895***	0.184	−0.001
	(0.148)	(0.104)	(0.062)	(0.164)	(0.104)	(0.057)
Canada (CA)	−0.284***	−0.239***	−0.246***	0.296***	0.337***	0.262****
	(0.064)	(0.072)	(0.072)	(0.061)	(0.060)	(0.078)
Switzerland (CH)	−0.268**	−0.471***	−0.731***	0.249**	0.057	−0.252**
	(0.085)	(0.070)	(0.083)	(0.082)	(0.075)	(0.094)
Australia (AU)	−0.739***	−0.619***	−0.401***	−0.117	0.024	−0.112
	(0.105)	(0.106)	(0.106)	(0.104)	(0.110)	(0.192)
Israel (IL)	−0.643***	−0.412**	−0.272*	−0.072	0.178	0.276*
	(0.128)	(0.129)	(0.115)	(0.119)	(0.134)	(0.122)
China (CN)	−1.967***	−1.526***	−0.363*	−1.409***	−0.973***	0.093
	(0.210)	(0.206)	(0.161)	(0.224)	(0.186)	(0.138)
India (IN)	−2.181***	−1.601***	−1.004***	−1.736***	−1.109***	−0.589*
	(0.214)	(0.197)	(0.206)	(0.167)	(0.208)	(0.266)
Former Soviet Union (FSU)	−1.398***	−1.238***	−0.900***	−0.892***	−0.739***	−0.481**
	(0.139)	(0.171)	(0.143)	(0.157)	(0.199)	(0.165)
Rest of the World (RW)	−0.729***	−0.537***	−0.416***	−0.143	0.081	0.144
	(0.073)	(0.080)	(0.086)	(0.075)	(0.079)	(0.080)

Notes. *p < 0.05, **p < 0.01, ***p < 0.001. The specifications are the same as for Table 2 except that here we include category dummies instead of sub-category dummies.

© 2017 The Authors.
The Economic Journal published by John Wiley & Sons Ltd on behalf of Royal Economic Society.

Table C2

Regression Results (log average citations, with five-year windows)

	Dependent variable					
	Log average citations			Log average citations (adjusted)		
Country	(1) 1980–9	(2) 1990–9	(3) 2000–6	(4) 1980–9	(5) 1990–9	(6) 2000–6
Japan (JP)	0.119**	−0.222***	−0.379***	0.285***	0.026	−0.147***
	(0.043)	(0.038)	(0.046)	(0.045)	(0.036)	(0.040)
Germany (DE)	−0.179***	−0.436***	−0.592***	0.226***	−0.003	−0.184***
	(0.037)	(0.032)	(0.043)	(0.035)	(0.035)	(0.041)
EU	−0.270***	−0.453***	−0.527***	0.235***	0.057	−0.052
	(0.036)	(0.035)	(0.059)	(0.035)	(0.043)	(0.068)
France (FR)	−0.265***	−0.473***	−0.636***	0.255***	0.042	−0.154**
	(0.036)	(0.036)	(0.044)	(0.036)	(0.042)	(0.051)
United Kingdom (UK)	−0.133**	−0.339***	−0.348***	0.403***	0.228***	0.195**
	(0.046)	(0.038)	(0.048)	(0.037)	(0.039)	(0.061)
Taiwan (TW)	−0.450***	−0.241***	−0.407***	0.050	0.158**	−0.045
	(0.055)	(0.062)	(0.064)	(0.066)	(0.053)	(0.049)
Korea (KR)	−0.891***	−0.485***	−0.475***	−0.351**	−0.017	−0.067
	(0.107)	(0.059)	(0.044)	(0.118)	(0.069)	(0.042)
Canada (CA)	−0.214***	−0.204***	−0.209***	0.344***	0.344***	0.292***
	(0.040)	(0.038)	(0.049)	(0.039)	(0.043)	(0.064)
Switzerland (CH)	−0.202***	−0.437***	−0.653***	0.277***	0.062	−0.161**
	(0.052)	(0.046)	(0.046)	(0.043)	(0.059)	(0.057)
Australia (AU)	−0.456***	−0.443***	−0.374***	0.159***	0.186**	0.028
	(0.039)	(0.045)	(0.064)	(0.048)	(0.060)	(0.124)
Israel (IL)	−0.319***	−0.250***	−0.141*	0.229**	0.346***	0.412***
	(0.082)	(0.054)	(0.071)	(0.070)	(0.073)	(0.082)
China (CN)	−1.041***	−1.064***	−0.458***	−0.416*	−0.468***	0.026
	(0.169)	(0.112)	(0.117)	(0.170)	(0.107)	(0.091)
India (IN)	−1.504***	−0.893***	−0.952***	−0.970***	−0.437*	−0.569**
	(0.123)	(0.133)	(0.132)	(0.159)	(0.182)	(0.182)
Former Soviet Union (FSU)	−1.026***	−0.879***	−0.750***	−0.461***	−0.297***	−0.226*
	(0.063)	(0.082)	(0.087)	(0.076)	(0.087)	(0.096)
Rest of the World (RW)	−0.542***	−0.494***	−0.442***	0.061	0.123*	0.112
	(0.037)	(0.042)	(0.056)	(0.043)	(0.055)	(0.060)

Notes. *p < 0.05, **p < 0.01, ***p < 0.001. The specifications are the same as for Table 2 except that here we use five year windows instead of two-year windows for forward citations. Note that the last sub-period includes patents granted up to only 2006 to reflect this. There are a total 14,009 observations. In the first (second) regression, zero average citations are replaced by 0.058 (0.060) in 472 (523) cells.

© 2017 The Authors.
The Economic Journal published by John Wiley & Sons Ltd on behalf of Royal Economic Society.

Table C3

Estimation Results of the Citation Lag Model for Chemical Sector

Country	Period 1 (1980–3)	2 (1984–7)	3 (1988–91)	4 (1992–5)	5 (1996–9)	6 (2000–3)	7 (2004–7)	8 (2008–11)
Japan (JP)	−0.15	−0.11	−0.14	−0.02	0.27	0.31	−0.04	0.46
	(0.05)	(0.05)	(0.05)	(0.05)	(0.05)	(0.06)	(0.09)	(0.26)
Germany (DE)	−0.32	−0.32	−0.12	−0.12	0.17	0.35	−0.02	−0.30
	(0.07)	(0.07)	(0.07)	(0.07)	(0.07)	(0.08)	(0.15)	(0.44)
EU	−0.16	−0.21	−0.16	−0.21	0.12	0.15	−0.35	0.52
	(0.07)	(0.07)	(0.07)	(0.07)	(0.07)	(0.08)	(0.35)	(0.37)
France (FR)	−0.12	−0.11	0.06	0.12	0.3	0.33	0.21	0.14
	(0.04)	(0.04)	(0.04)	(0.03)	(0.04)	(0.04)	(0.07)	(0.23)
United Kingdom (UK)	−0.34	−0.45	−0.34	−0.15	0.18	0.11	−0.15	0.72
	(0.06)	(0.06)	(0.06)	(0.05)	(0.06)	(0.06)	(0.11)	(0.42)
Taiwan (TW)	−1.12	−1.26	−0.64	−0.19	0.16	0.18	−0.13	0.1
	(0.29)	(0.23)	(0.16)	(0.11)	(0.09)	(0.08)	(0.12)	(0.31)
Korea (KR)	−0.48	−0.92	−0.29	−0.17	0.39	0.5	0.38	0.15
	(0.23)	(0.23)	(0.14)	(0.11)	(0.09)	(0.08)	(0.13)	(0.30)
Canada (CA)	−0.26	−0.4	−0.54	−0.29	−0.08	−0.25	−0.46	−1.02
	(0.16)	(0.17)	(0.3)	(0.23)	(0.21)	(0.27)	(0.24)	(0.72)
Switzerland (CH)	−0.47	−0.41	−0.11	−0.42	−0.22	−0.08	−0.14	−0.84
	(0.08)	(0.07)	(0.2)	(0.16)	(0.16)	(0.18)	(0.25)	(0.72)
Australia (AU)	−0.1	−0.31	−0.28	−0.19	0.12	0.13	−0.61	0.08
	(0.09)	(0.11)	(0.07)	(0.06)	(0.07)	(0.08)	(0.13)	(0.33)
Israel (IL)	−0.39	−0.61	0.01	−0.06	0.24	0.33	−0.17	−0.24
	(0.21)	(0.2)	(0.11)	(0.11)	(0.11)	(0.13)	(0.21)	(0.52)
China (CN)			−0.48	−0.46	−0.11	0.38	−0.77	0.93
			(0.17)	(0.17)	(0.17)	(0.17)	(0.27)	(0.62)
India (IN)			−0.69	−0.44	0.17	−0.11	−0.71	
			(0.31)	(0.25)	(0.2)	(0.2)	(0.37)	
Former Soviet Union (FSU)	−0.24	−1.28	−0.43	0	0.29	−0.13	0.14	
	(0.25)	(0.33)	(0.26)	(0.22)	(0.25)	(0.24)	(0.39)	
Rest of the World (RW)	−0.51	−0.49	−0.32	−0.5	−0.09	0.14	−0.32	0.00
	(0.1)	(0.11)	(0.12)	(0.1)	(0.1)	(0.11)	(0.17)	(0.45)

Notes. The specifications are the same as for Table 4 except that here only chemical sector patents are considered among the cited patents. We include CN and IN in RW for the first two sub-periods to avoid diverging estimators due to their small sample sizes. For the same reason, IN and FSU are included in RW for the last sub-period.

© 2017 The Authors.
The Economic Journal published by John Wiley & Sons Ltd on behalf of Royal Economic Society.

Table C4

Estimation Results of the Citation Lag Model for Computers and Communications Sector

	Period							
Country	1 (1980–3)	2 (1984–7)	3 (1988–91)	4 (1992–5)	5 (1996–9)	6 (2000–3)	7 (2004–7)	8 (2008–11)
Japan (JP)	−0.34	−0.55	−0.83	−0.95	−0.4	−0.54	−0.4	−0.27
	(0.1)	(0.08)	(0.08)	(0.07)	(0.05)	(0.04)	(0.05)	(0.11)
Germany (DE)	−0.76	−0.94	−0.79	−0.92	−0.32	−0.63	−0.46	−0.29
	(0.11)	(0.09)	(0.09)	(0.08)	(0.06)	(0.06)	(0.06)	(0.13)
EU	−0.69	−0.82	−0.86	−0.89	−0.53	−0.62	−0.66	−0.32
	(0.11)	(0.09)	(0.09)	(0.08)	(0.06)	(0.05)	(0.05)	(0.13)
France (FR)	−0.5	−0.35	−0.28	−0.36	−0.13	−0.3	−0.19	−0.06
	(0.05)	(0.04)	(0.04)	(0.03)	(0.02)	(0.02)	(0.03)	(0.07)
United Kingdom (UK)	−0.93	−0.83	−1	−1.16	−0.41	−0.59	−0.48	−0.44
	(0.12)	(0.1)	(0.09)	(0.07)	(0.05)	(0.04)	(0.05)	(0.14)
Taiwan (TW)	−2.65	−1.96	−1.29	−1	−0.24	−0.5	−0.4	−0.18
	(0.63)	(0.33)	(0.16)	(0.09)	(0.05)	(0.04)	(0.05)	(0.1)
Korea (KR)	−2.2	−1.41	−1.16	−0.98	−0.2	−0.3	−0.24	−0.28
	(0.39)	(0.26)	(0.16)	(0.11)	(0.07)	(0.05)	(0.05)	(0.11)
Canada (CA)	−0.85	−1.27	−2.76	−0.93	−0.79	−1.06	−0.95	−0.23
	(0.29)	(0.28)	(0.96)	(0.52)	(0.32)	(0.16)	(0.09)	(0.15)
Switzerland (CH)	−0.49	−0.77	−1.03	−0.93	−0.37	−0.72	−0.78	−0.51
	(0.14)	(0.11)	(0.27)	(0.22)	(0.13)	(0.1)	(0.1)	(0.21)
Australia (AU)	−0.58	−0.25	−1.12	−0.99	−0.42	−0.61	−0.61	−0.45
	(0.21)	(0.22)	(0.1)	(0.08)	(0.05)	(0.04)	(0.05)	(0.1)
Israel (IL)	−1.11	−1.3	−0.85	−1.11	−0.41	−0.79	−0.38	0.08
	(0.32)	(0.25)	(0.19)	(0.18)	(0.15)	(0.11)	(0.13)	(0.27)
China (CN)			−1.55	−0.9	−0.61	−0.75	−0.69	−0.62
			(0.17)	(0.12)	(0.07)	(0.06)	(0.07)	(0.14)
India (IN)			−1.42	0	−0.67	−0.86	−0.72	−0.17
			(0.82)	(0.45)	(0.25)	(0.11)	(0.1)	(0.14)
Former Soviet Union (FSU)	0.94	−1.98	−1.61	−1.53	0.69	−1.16	−0.8	0.22
	(0.62)	(0.64)	(0.61)	(0.34)	(0.25)	(0.23)	(0.19)	(0.29)
Rest of the World (RW)	−1.29	−1.23	−1.15	−0.88	−0.61	−0.62	−0.79	−0.59
	(0.24)	(0.22)	(0.21)	(0.15)	(0.09)	(0.07)	(0.08)	(0.16)

Notes. The specifications are the same as for Table 4 except that only computers and communications sector patents are considered among the cited patents. We include CN and IN in RW for the first two sub-periods to avoid diverging estimators due to their small sample sizes.

© 2017 The Authors.
The Economic Journal published by John Wiley & Sons Ltd on behalf of Royal Economic Society.

Table C5

Estimation Results of the Citation Lag Model for Drugs and Medical Sector

	Period							
Country	1 (1980–3)	2 (1984–7)	3 (1988–91)	4 (1992–95)	5 (1996–9)	6 (2000–3)	7 (2004–7)	8 (2008–11)
Japan (JP)	−0.4	−0.46	−0.35	−0.74	0.4	0.22	−0.16	0.18
	(0.1)	(0.09)	(0.07)	(0.07)	(0.06)	(0.06)	(0.13)	(0.24)
Germany (DE)	−0.59	−0.62	−0.56	−0.73	0.35	0.17	−0.24	−0.18
	(0.13)	(0.11)	(0.1)	(0.09)	(0.08)	(0.08)	(0.16)	(0.36)
EU	−0.66	−0.66	−0.41	−0.63	0.33	0.28	−0.19	−0.37
	(0.12)	(0.11)	(0.09)	(0.09)	(0.07)	(0.08)	(0.17)	(0.35)
France (FR)	−0.45	−0.38	−0.28	−0.42	0.43	0.38	−0.08	−0.09
	(0.08)	(0.07)	(0.06)	(0.07)	(0.05)	(0.05)	(0.12)	(0.32)
United Kingdom (UK)	−0.67	−0.74	−0.41	−0.75	0.26	0.1	−0.4	−0.64
	(0.1)	(0.09)	(0.08)	(0.07)	(0.05)	(0.06)	(0.12)	(0.31)
Taiwan (TW)	−2.53	−1.52	−0.57	−0.87	0.49	0	−0.21	−0.38
	(1.31)	(0.55)	(0.33)	(0.22)	(0.14)	(0.12)	(0.28)	(0.64)
Korea (KR)	−1.45	−0.65	−0.09	−0.6	0.19	0.29	−0.32	−0.94
	(0.49)	(0.38)	(0.23)	(0.18)	(0.12)	(0.12)	(0.26)	(0.64)
Canada (CA)	−0.93	−0.26	−0.8	−1.34	−0.19	−0.43	−1.18	0.26
	(0.26)	(0.2)	(0.44)	(0.47)	(0.24)	(0.22)	(0.59)	(0.61)
Switzerland (CH)	−0.79	−0.81	−0.57	−0.86	0.25	0.09	−0.4	0.27
	(0.15)	(0.13)	(0.17)	(0.15)	(0.11)	(0.12)	(0.33)	(0.52)
Australia (AU)	−0.36	−0.29	−0.73	−0.9	0.18	0.09	−0.51	0.43
	(0.17)	(0.16)	(0.1)	(0.09)	(0.07)	(0.07)	(0.15)	(0.36)
Israel (IL)	−0.66	−0.85	−0.73	−0.84	0.54	0.24	−0.47	0.21
	(0.24)	(0.21)	(0.15)	(0.14)	(0.11)	(0.13)	(0.2)	(0.33)
China (CN)			−0.38	−0.88	−0.11	−0.05	−0.67	−0.14
			(0.16)	(0.13)	(0.09)	(0.11)	(0.19)	(0.31)
India (IN)			−0.4	−1.28	0.28	0.43	−0.53	−0.63
			(0.46)	(0.32)	(0.19)	(0.2)	(0.54)	(1.5)
Former Soviet Union (FSU)	−0.72	−0.52	−1.02	−0.43	−0.09	0.19	−0.57	−3.23
	(0.41)	(0.3)	(0.38)	(0.33)	(0.21)	(0.23)	(0.61)	(1.43)
Rest of the World (RW)	−0.8	−1.08	−0.6	−0.96	0.05	0.11	−0.64	−0.1
	(0.18)	(0.18)	(0.14)	(0.13)	(0.11)	(0.11)	(0.21)	(0.34)

Notes. The specifications are the same as for Table 4 except that only drugs and medical sector patents are considered among the cited patents. We include CN and IN in RW for the first two sub-periods to avoid diverging estimators due to their small sample sizes.

© 2017 The Authors.
The Economic Journal published by John Wiley & Sons Ltd on behalf of Royal Economic Society.

Table C6

Estimation Results of the Citation Lag Model for Electrical and Electronics Sector

Country	Period 1 (1980–3)	2 (1984–7)	3 (1988–91)	4 (1992–5)	5 (1996–9)	6 (2000–3)	7 (2004–7)	8 (2008–11)
Japan (JP)	−0.42	−0.2	−0.25	−0.28	−0.24	0.01	−0.01	0.03
	(0.05)	(0.05)	(0.05)	(0.05)	(0.04)	(0.04)	(0.06)	(0.12)
Germany (DE)	−0.41	−0.35	−0.19	−0.2	−0.25	0.01	0.09	−0.3
	(0.07)	(0.06)	(0.06)	(0.07)	(0.07)	(0.06)	(0.1)	(0.2)
EU	−0.49	−0.28	−0.28	−0.4	−0.21	0	−0.02	−0.32
	(0.07)	(0.07)	(0.07)	(0.07)	(0.07)	(0.06)	(0.1)	(0.34)
France (FR)	−0.33	−0.11	−0.07	−0.08	0.05	0.11	0.11	0.09
	(0.04)	(0.03)	(0.03)	(0.03)	(0.03)	(0.02)	(0.03)	(0.09)
United Kingdom (UK)	−0.64	−0.4	−0.4	−0.31	−0.33	−0.13	−0.19	−0.06
	(0.07)	(0.06)	(0.06)	(0.06)	(0.05)	(0.05)	(0.06)	(0.15)
Taiwan (TW)	−1.71	−1.07	−0.47	−0.33	−0.04	−0.02	0	0.07
	(0.22)	(0.16)	(0.1)	(0.07)	(0.05)	(0.04)	(0.05)	(0.12)
Korea (KR)	−1.22	−0.87	−0.52	−0.29	−0.02	0.19	0.11	0.24
	(0.17)	(0.13)	(0.09)	(0.06)	(0.04)	(0.03)	(0.05)	(0.12)
Canada (CA)	−0.41	−0.71	−0.91	−1.32	−0.77	−0.44	−0.37	−0.05
	(0.18)	(0.18)	(0.41)	(0.35)	(0.28)	(0.13)	(0.1)	(0.15)
Switzerland (CH)	−0.63	−0.44	−0.71	−0.57	−0.33	−0.49	0.07	0.4
	(0.09)	(0.08)	(0.19)	(0.21)	(0.19)	(0.15)	(0.25)	(0.9)
Australia (AU)	−0.35	−0.42	−0.45	−0.5	−0.34	−0.07	−0.21	−0.23
	(0.11)	(0.11)	(0.07)	(0.07)	(0.06)	(0.06)	(0.09)	(0.19)
Israel (IL)	−1.3	−1.03	−0.38	−0.39	−0.13	−0.24	0.13	0.22
	(0.27)	(0.17)	(0.12)	(0.12)	(0.15)	(0.11)	(0.14)	(0.28)
China (CN)			−0.55	−0.58	−0.32	−0.28	−0.14	0.28
			(0.13)	(0.13)	(0.11)	(0.1)	(0.12)	(0.34)
India (IN)			−1.7	−0.79	−1.25	−0.66	−0.24	−0.65
			(0.66)	(0.47)	(0.31)	(0.15)	(0.16)	(0.3)
Former Soviet Union (FSU)	−0.21	−0.53	−0.05	−0.11	−0.26	−0.06	−0.16	−1.89
	(0.28)	(0.36)	(0.31)	(0.3)	(0.2)	(0.18)	(0.28)	(1.29)
Rest of the World (RW)	−0.86	−0.8	−0.74	−0.61	−0.36	−0.12	−0.3	0.13
	(0.15)	(0.13)	(0.13)	(0.11)	(0.08)	(0.06)	(0.09)	(0.19)

Notes. The specifications are the same as for Table 4 except that only electrical and electronics sector patents are considered among the cited patents. We include CN and IN in RW for the first two sub-periods to avoid diverging estimators due to their small sample sizes.

© 2017 The Authors.
The Economic Journal published by John Wiley & Sons Ltd on behalf of Royal Economic Society.

Table C7

Estimation Results of the Citation Lag Model for Mechanical Sector

	Period							
Country	1 (1980–3)	2 (1984–7)	3 (1988–91)	4 (1992–5)	5 (1996–9)	6 (2000–3)	7 (2004–7)	8 (2008–11)
Japan (JP)	−0.31	−0.15	−0.06	−0.18	0.04	0.18	−0.05	0.03
	(0.04)	(0.04)	(0.04)	(0.04)	(0.04)	(0.04)	(0.09)	(0.15)
Germany (DE)	−0.39	−0.24	0.03	−0.21	−0.01	0.16	−0.14	−0.27
	(0.07)	(0.06)	(0.07)	(0.07)	(0.07)	(0.08)	(0.13)	(0.44)
EU	−0.27	−0.14	−0.08	−0.29	−0.04	0.09	−0.09	0.21
	(0.06)	(0.06)	(0.07)	(0.07)	(0.07)	(0.08)	(0.12)	(0.28)
France (FR)	−0.28	−0.1	0.07	0	0.1	0.23	0.19	0.13
	(0.04)	(0.03)	(0.03)	(0.03)	(0.03)	(0.03)	(0.06)	(0.12)
United Kingdom (UK)	−0.46	−0.24	−0.17	−0.27	−0.03	0.06	0.07	−0.42
	(0.06)	(0.05)	(0.06)	(0.05)	(0.05)	(0.05)	(0.09)	(0.24)
Taiwan (TW)	−1.19	−0.81	−0.59	−0.39	0.07	0.23	0.15	0.16
	(0.23)	(0.2)	(0.15)	(0.1)	(0.08)	(0.09)	(0.1)	(0.23)
Korea (KR)	−1.12	−0.63	−0.39	−0.17	0.09	0.23	0.23	−0.09
	(0.14)	(0.1)	(0.09)	(0.07)	(0.06)	(0.05)	(0.09)	(0.18)
Canada (CA)	−0.52	−0.45	−1.52	−1	−1.07	−0.38	−0.21	−0.43
	(0.13)	(0.13)	(0.47)	(0.37)	(0.35)	(0.19)	(0.21)	(0.32)
Switzerland (CH)	−0.5	−0.4	−0.46	−0.38	−0.07	−0.22	−0.38	−0.52
	(0.07)	(0.06)	(0.13)	(0.13)	(0.13)	(0.12)	(0.22)	(0.63)
Australia (AU)	−0.33	−0.21	−0.26	−0.29	−0.1	0.09	−0.24	−0.15
	(0.09)	(0.09)	(0.06)	(0.05)	(0.05)	(0.05)	(0.1)	(0.21)
Israel (IL)	−0.89	−0.49	0.02	−0.35	−0.08	0.18	−0.34	1.13
	(0.19)	(0.17)	(0.1)	(0.11)	(0.1)	(0.11)	(0.23)	(0.58)
China (CN)			−0.08	−0.34	−0.12	0.1	−0.23	−0.36
			(0.15)	(0.15)	(0.15)	(0.15)	(0.21)	(0.57)
India (IN)			−1.21	1.4	−0.89	−0.61	−0.97	
			(0.82)	(1.12)	(0.48)	(0.42)	(0.79)	
Former Soviet Union (FSU)	−0.5	−0.53	−0.35	−0.28	0.3	0.1	−0.77	
	(0.27)	(0.32)	(0.27)	(0.25)	(0.28)	(0.27)	(0.86)	
Rest of the World (RW)	−0.68	−0.3	−0.47	−0.37	−0.14	−0.15	0.01	−0.18
	(0.11)	(0.11)	(0.12)	(0.1)	(0.09)	(0.09)	(0.14)	(0.32)

Notes. The specifications are the same as for Table 4 except that only mechanical sector patents are considered among the cited patents. We include CN and IN in RW for the first two sub-periods to avoid diverging estimators due to their small sample sizes. For the same reason, IN and FSU are included in RW for the last sub-period.

© 2017 The Authors.
The Economic Journal published by John Wiley & Sons Ltd on behalf of Royal Economic Society.

Table C8

Estimation Results of the Citation Lag Model for Other Sectors

	Period							
Country	1 (1980–3)	2 (1984–7)	3 (1988–91)	4 (1992–5)	5 (1996–9)	6 (2000–3)	7 (2004–7)	8 (2008–11)
Japan (JP)	−0.14	−0.1	0.05	−0.11	0.16	0.31	−0.11	0.19
	(0.05)	(0.04)	(0.05)	(0.05)	(0.04)	(0.05)	(0.08)	(0.22)
Germany (DE)	−0.13	−0.09	0	−0.07	0.09	0.32	0.01	−0.87
	(0.06)	(0.06)	(0.06)	(0.07)	(0.07)	(0.07)	(0.12)	(0.37)
EU	−0.25	−0.11	−0.22	−0.19	0.1	0.21	−0.14	0.09
	(0.06)	(0.06)	(0.06)	(0.06)	(0.06)	(0.06)	(0.1)	(0.24)
France (FR)	−0.17	0.04	0.12	−0.01	0.27	0.38	0.23	0.31
	(0.04)	(0.04)	(0.04)	(0.04)	(0.04)	(0.04)	(0.06)	(0.17)
United Kingdom (UK)	−0.35	−0.15	−0.14	−0.16	0.13	0.33	0.13	0.06
	(0.05)	(0.05)	(0.05)	(0.05)	(0.05)	(0.05)	(0.08)	(0.2)
Taiwan (TW)	−0.92	−0.99	−0.36	−0.33	0.11	0.16	−0.05	0.27
	(0.19)	(0.18)	(0.13)	(0.1)	(0.08)	(0.1)	(0.11)	(0.26)
Korea (KR)	−0.73	−0.55	−0.15	−0.04	0.26	0.44	0.08	0.48
	(0.12)	(0.08)	(0.06)	(0.06)	(0.05)	(0.05)	(0.08)	(0.22)
Canada (CA)	−0.58	−0.16	0.22	−1.37	−0.31	−0.14	−0.26	0.54
	(0.1)	(0.1)	(0.36)	(0.37)	(0.22)	(0.18)	(0.17)	(0.3)
Switzerland (CH)	−0.42	−0.31	−0.25	−0.25	0.05	−0.01	−0.25	0.41
	(0.05)	(0.05)	(0.1)	(0.1)	(0.09)	(0.11)	(0.18)	(0.42)
Australia (AU)	−0.29	−0.04	−0.22	−0.21	0.07	0.1	−0.24	−0.1
	(0.09)	(0.09)	(0.04)	(0.04)	(0.04)	(0.04)	(0.07)	(0.24)
Israel (IL)	−0.68	−0.38	0.08	−0.03	0.26	0.17	0.07	0.54
	(0.16)	(0.16)	(0.1)	(0.11)	(0.1)	(0.13)	(0.19)	(0.75)
China (CN)			−0.27	−0.21	0.07	0.51	−0.51	−0.13
			(0.14)	(0.14)	(0.13)	(0.18)	(0.23)	(0.56)
India (IN)			−0.8	−2.08	−0.44	−0.53	0.52	
			(0.7)	(0.68)	(0.41)	(0.33)	(0.73)	
Former Soviet Union (FSU)	0.18	−0.59	0.08	−0.82	−0.04	0.07	0.04	
	(0.31)	(0.31)	(0.32)	(0.34)	(0.32)	(0.29)	(0.44)	
Rest of the World (RW)	−0.4	−0.45	−0.3	−0.43	−0.04	0.04	−0.04	0.02
	(0.09)	(0.08)	(0.09)	(0.08)	(0.07)	(0.07)	(0.1)	(0.26)

Notes. The specifications are the same as for Table 4 except that only patents not categorised as the aforementioned five sectors are considered among the cited patents. We include CN and IN in RW for the first two sub-periods to avoid diverging estimators due to their small sample sizes. For the same reason, IN and FSU are included in RW for the last sub-period.

© 2017 The Authors.
The Economic Journal published by John Wiley & Sons Ltd on behalf of Royal Economic Society.

Table C9

Estimation Results of the Citation Lag Model with the Second and Third Citations

	Period							
Country	1 (1980–3)	2 (1984–7)	3 (1988–91)	4 (1992–5)	5 (1996–9)	6 (2000–3)	7 (2004–7)	8 (2008–11)
Japan (JP)	−0.11	−0.04	0.01	−0.03	0.11	0.04	−0.02	0.18
	(0.02)	(0.02)	(0.01)	(0.01)	(0.01)	(0.01)	(0.02)	(0.06)
Germany (DE)	−0.17	−0.11	−0.14	−0.15	0.02	−0.07	−0.12	0.1
	(0.02)	(0.02)	(0.02)	(0.02)	(0.02)	(0.02)	(0.03)	(0.09)
EU	−0.26	−0.24	−0.22	−0.28	0	−0.14	−0.18	−0.03
	(0.03)	(0.03)	(0.03)	(0.02)	(0.02)	(0.02)	(0.03)	(0.1)
France (FR)	−0.2	−0.15	−0.13	−0.12	0.03	−0.07	−0.16	0.16
	(0.03)	(0.03)	(0.03)	(0.03)	(0.03)	(0.03)	(0.05)	(0.13)
United Kingdom (UK)	−0.22	−0.12	−0.17	−0.26	−0.05	−0.14	−0.16	0.12
	(0.03)	(0.03)	(0.03)	(0.03)	(0.03)	(0.03)	(0.04)	(0.12)
Taiwan (TW)	−0.69	−0.47	−0.33	−0.27	0.09	0.05	−0.03	0.13
	(0.07)	(0.05)	(0.04)	(0.03)	(0.03)	(0.02)	(0.03)	(0.1)
Korea (KR)	−0.72	−0.71	−0.47	−0.28	0	−0.13	−0.13	−0.16
	(0.1)	(0.08)	(0.05)	(0.04)	(0.03)	(0.03)	(0.03)	(0.09)
Canada (CA)	−0.42	−0.31	−0.23	−0.26	−0.04	−0.14	−0.23	0.05
	(0.03)	(0.03)	(0.03)	(0.03)	(0.02)	(0.02)	(0.04)	(0.1)
Switzerland (CH)	−0.15	−0.09	−0.14	−0.14	−0.04	−0.07	−0.22	0.14
	(0.05)	(0.05)	(0.05)	(0.05)	(0.05)	(0.06)	(0.09)	(0.29)
Australia (AU)	−0.33	−0.39	−0.32	−0.38	−0.22	−0.27	−0.3	0.08
	(0.07)	(0.06)	(0.06)	(0.06)	(0.05)	(0.05)	(0.08)	(0.23)
Israel (IL)	−0.36	−0.29	−0.32	−0.35	−0.13	−0.18	−0.29	0.13
	(0.09)	(0.07)	(0.06)	(0.05)	(0.04)	(0.04)	(0.06)	(0.13)
China (CN)			−0.94	−0.54	−0.4	−0.43	−0.28	−0.11
			(0.16)	(0.13)	(0.1)	(0.07)	(0.06)	(0.12)
India (IN)			−0.9	−0.65	−0.21	−0.27	−0.32	−0.32
			(0.2)	(0.15)	(0.12)	(0.08)	(0.08)	(0.17)
Former Soviet Union (FSU)	−0.2	−0.23	−0.51	−0.41	0.1	−0.12	−0.05	0.09
	(0.15)	(0.14)	(0.14)	(0.11)	(0.1)	(0.11)	(0.14)	(0.38)
Rest of the World (RW)	−0.35	−0.34	−0.31	−0.34	−0.19	−0.18	−0.26	−0.17
	(0.05)	(0.05)	(0.05)	(0.04)	(0.04)	(0.03)	(0.05)	(0.21)

Notes. The reported numbers are the estimated coefficient values for the country dummy estimated from the regression results above. In the parentheses are standard errors. Our regression model controls for base cohort size (the number of patents in the citing country and technology sub-category for the citing year), self citations (citations between patents with identical assignees) and within-sub-category citations (citations between patents within the same category). Corporation dummies (whether or not the first assignee is a corporation) and category dummies are also included. We include CN and IN in RW for the first two periods to avoid diverging estimators due to the small sample sizes of such cohorts.

© 2017 The Authors.

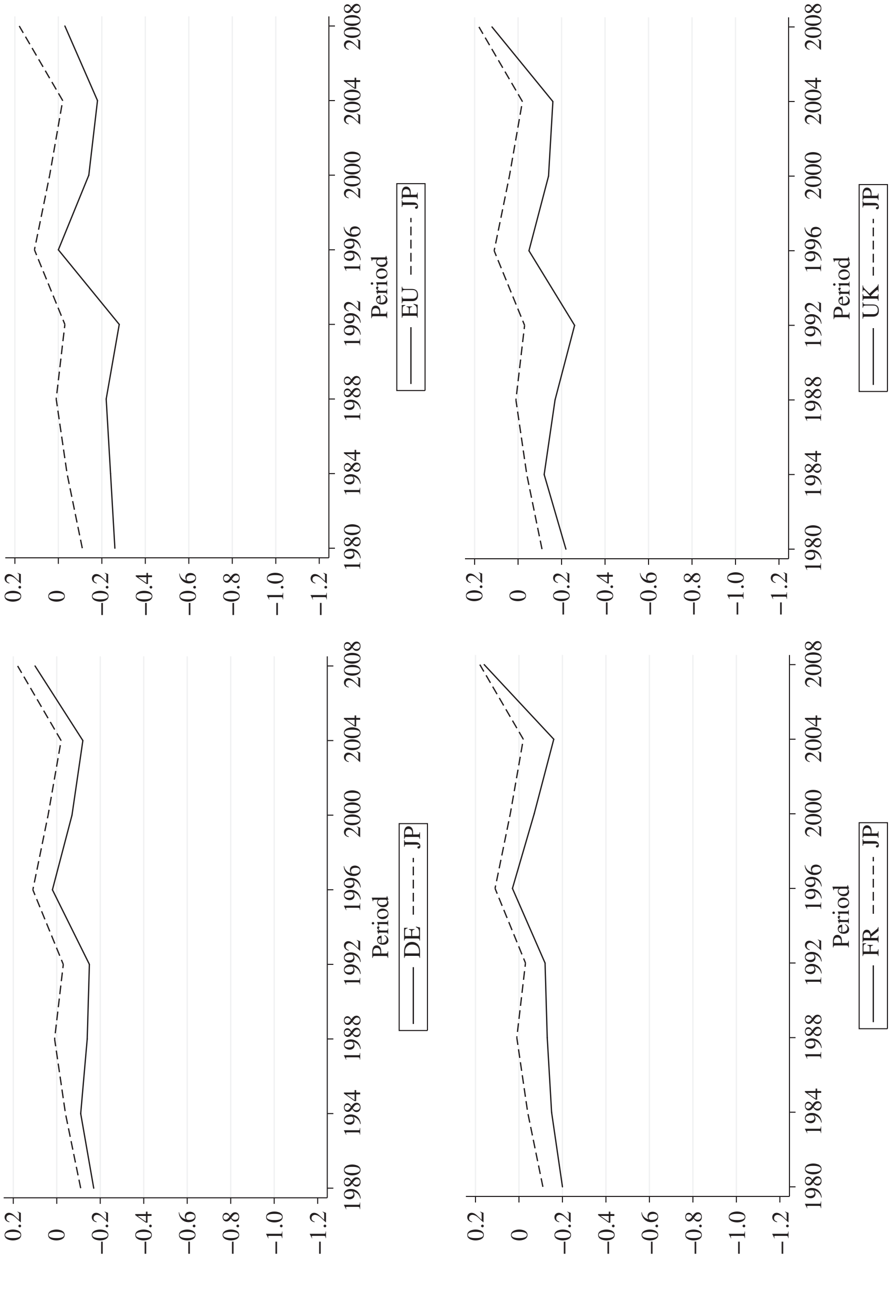

Fig. C1. *Graphical Representation of Estimation Results in Table C9*

Notes. Each graph plots the coefficient values for the country dummy. Japan is included in every graph as a benchmark.

© 2017 The Authors.
The Economic Journal published by John Wiley & Sons Ltd on behalf of Royal Economic Society.

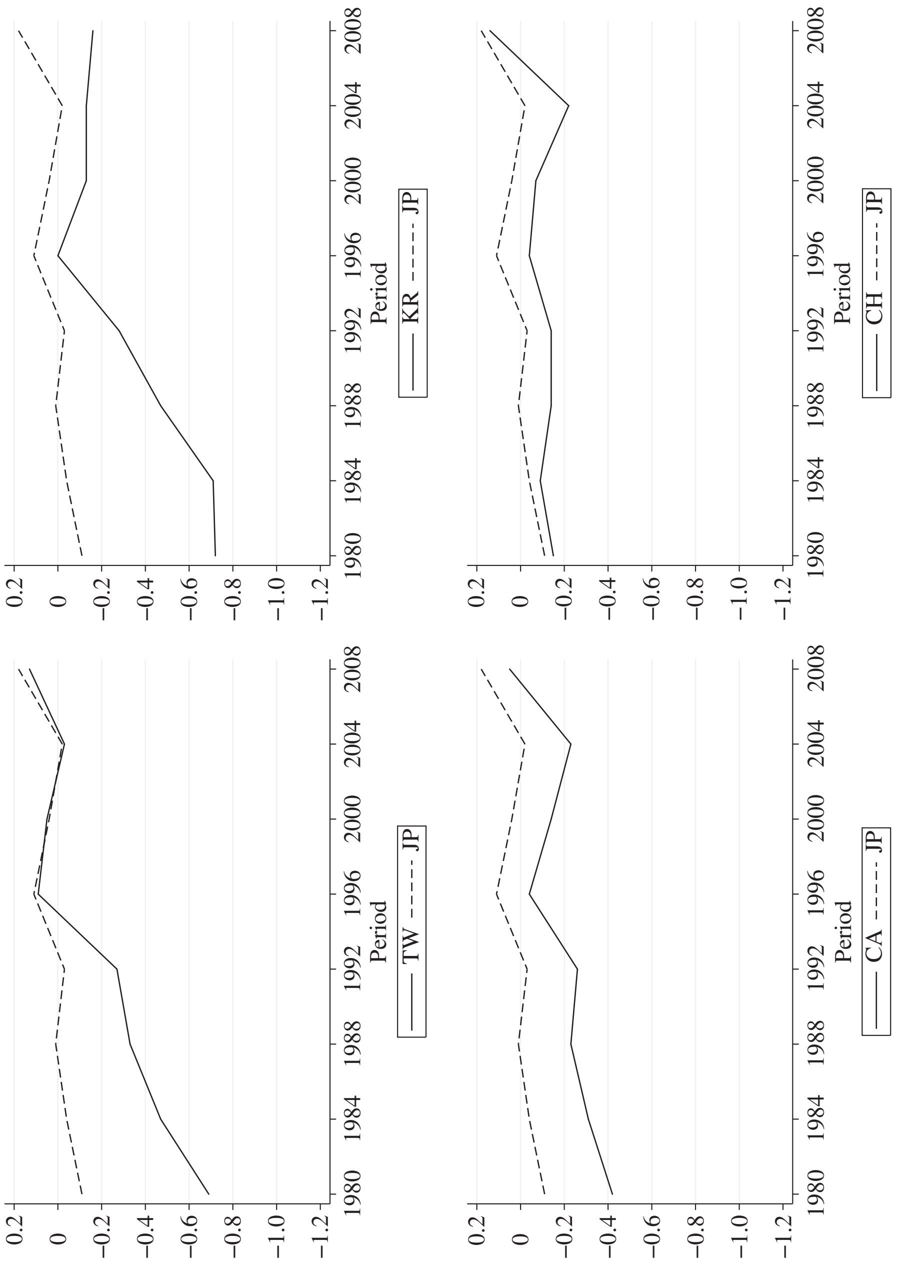

Fig. C1. *(Continued)*

© 2017 The Authors.
The Economic Journal published by John Wiley & Sons Ltd on behalf of Royal Economic Society.

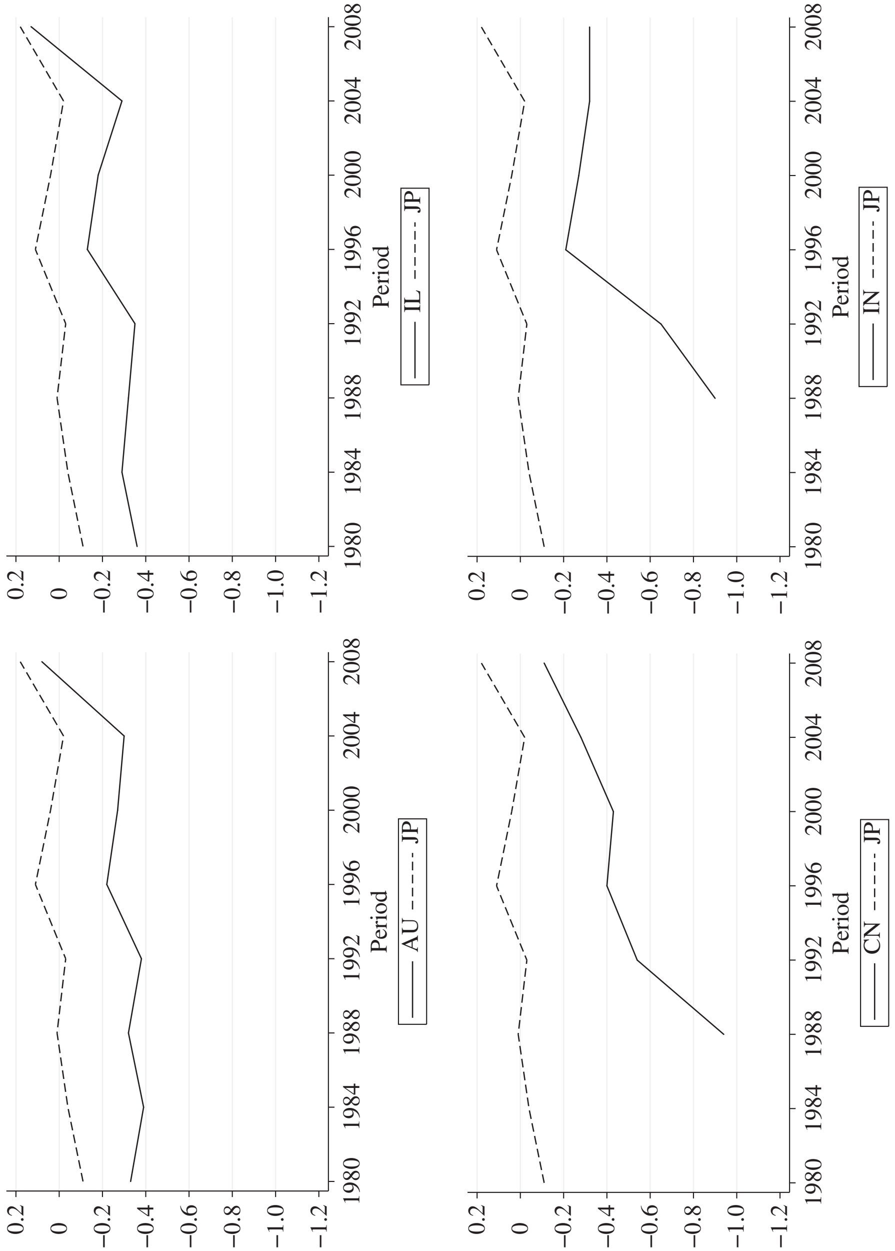

Fig. C1. *(Continued)*

© 2017 The Authors.
The Economic Journal published by John Wiley & Sons Ltd on behalf of Royal Economic Society.

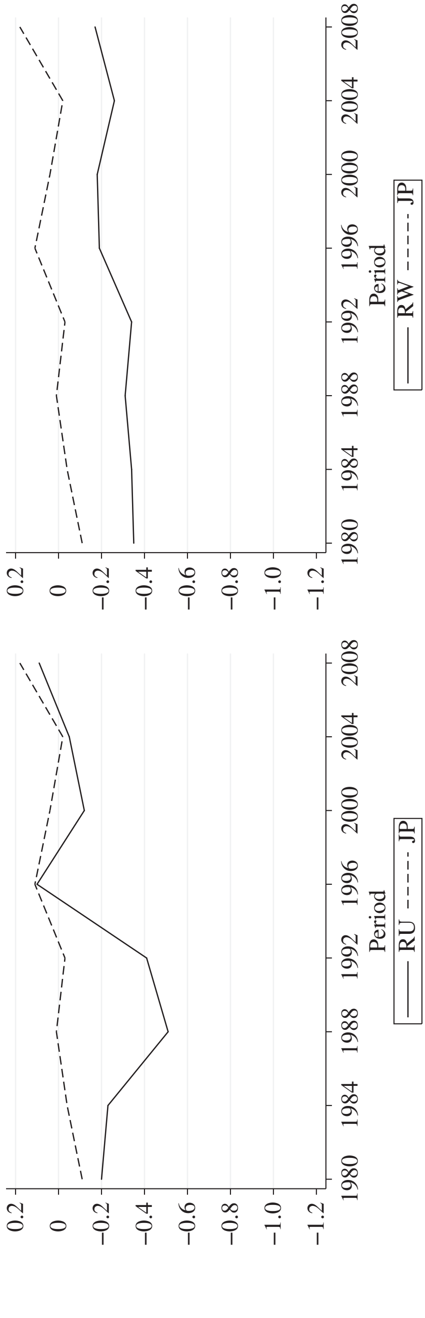

Fig. C1. *(Continued)*

© 2017 The Authors.
The Economic Journal published by John Wiley & Sons Ltd on behalf of Royal Economic Society.

Table C10

Estimation Results of the Citation Lag Model with the Third and Fourth Citations

	Period							
Country	1 (1980–3)	2 (1984–7)	3 (1988–91)	4 (1992–5)	5 (1996–9)	6 (2000–3)	7 (2004–7)	8 (2008–11)
Japan (JP)	−0.13	−0.03	0.01	0.01	0.02	0.01	0	0.13
	(0.02)	(0.02)	(0.02)	(0.02)	(0.01)	(0.02)	(0.03)	(0.09)
Germany (DE)	−0.16	−0.1	−0.11	−0.12	−0.03	−0.12	−0.08	0.14
	(0.03)	(0.02)	(0.02)	(0.02)	(0.02)	(0.03)	(0.04)	(0.14)
EU	−0.27	−0.22	−0.21	−0.15	−0.08	−0.16	−0.1	0
	(0.03)	(0.03)	(0.03)	(0.03)	(0.02)	(0.03)	(0.04)	(0.13)
France (FR)	−0.23	−0.09	−0.18	−0.16	−0.03	−0.1	0.01	−0.15
	(0.03)	(0.03)	(0.03)	(0.03)	(0.03)	(0.03)	(0.06)	(0.19)
United Kingdom (UK)	−0.2	−0.17	−0.16	−0.16	−0.08	−0.14	−0.14	−0.16
	(0.03)	(0.03)	(0.03)	(0.04)	(0.03)	(0.04)	(0.05)	(0.17)
Taiwan (TW)	−0.51	−0.33	−0.29	−0.18	−0.01	−0.06	−0.08	0.09
	(0.06)	(0.05)	(0.04)	(0.04)	(0.03)	(0.03)	(0.06)	(0.15)
Korea (KR)	−0.84	−0.54	−0.35	−0.18	−0.08	−0.16	−0.02	0.2
	(0.09)	(0.07)	(0.06)	(0.04)	(0.03)	(0.03)	(0.04)	(0.13)
Canada (CA)	−0.34	−0.23	−0.25	−0.17	−0.08	−0.11	−0.1	0.07
	(0.04)	(0.03)	(0.03)	(0.03)	(0.02)	(0.03)	(0.05)	(0.13)
Switzerland (CH)	−0.18	−0.12	−0.17	−0.21	−0.08	−0.15	−0.27	−0.19
	(0.07)	(0.08)	(0.06)	(0.06)	(0.05)	(0.06)	(0.13)	(0.33)
Australia (AU)	−0.31	−0.32	−0.28	−0.3	−0.09	−0.3	−0.26	−0.2
	(0.07)	(0.08)	(0.06)	(0.06)	(0.07)	(0.06)	(0.09)	(0.25)
Israel (IL)	−0.39	−0.32	−0.27	−0.25	−0.1	−0.27	−0.21	0.15
	(0.09)	(0.08)	(0.07)	(0.05)	(0.05)	(0.04)	(0.07)	(0.18)
China (CN)			−0.44	−0.77	−0.48	−0.35	−0.31	0.21
			(0.19)	(0.14)	(0.1)	(0.07)	(0.08)	(0.2)
India (IN)			−1.15	−0.65	−0.23	−0.49	−0.27	−0.32
			(0.28)	(0.17)	(0.13)	(0.09)	(0.09)	(0.21)
Former Soviet Union (FSU)	−0.45	−0.1	−0.13	−0.29	−0.04	−0.22	−0.23	−0.74
	(0.16)	(0.17)	(0.14)	(0.12)	(0.12)	(0.13)	(0.18)	(0.66)
Rest of the World (RW)	−0.54	−0.34	−0.33	−0.2	−0.04	−0.09	−0.19	−0.25
	(0.06)	(0.06)	(0.05)	(0.04)	(0.04)	(0.04)	(0.07)	(0.2)

Notes. The reported numbers are the estimated coefficient values for the country dummy estimated from the regression results above. In the parentheses are standard errors. Our regression model controls for base cohort size (the number of patents in the citing country and technology sub-category for the citing year), self citations (citations between patents with identical assignees) and within-sub-category citations (citations between patents within the same category). Corporation dummies (whether or not the first assignee is a corporation) and category dummies are also included. We include CN and IN in RW for the first two periods to avoid diverging estimators due to the small sample sizes of such cohorts.

© 2017 The Authors.
The Economic Journal published by John Wiley & Sons Ltd on behalf of Royal Economic Society.

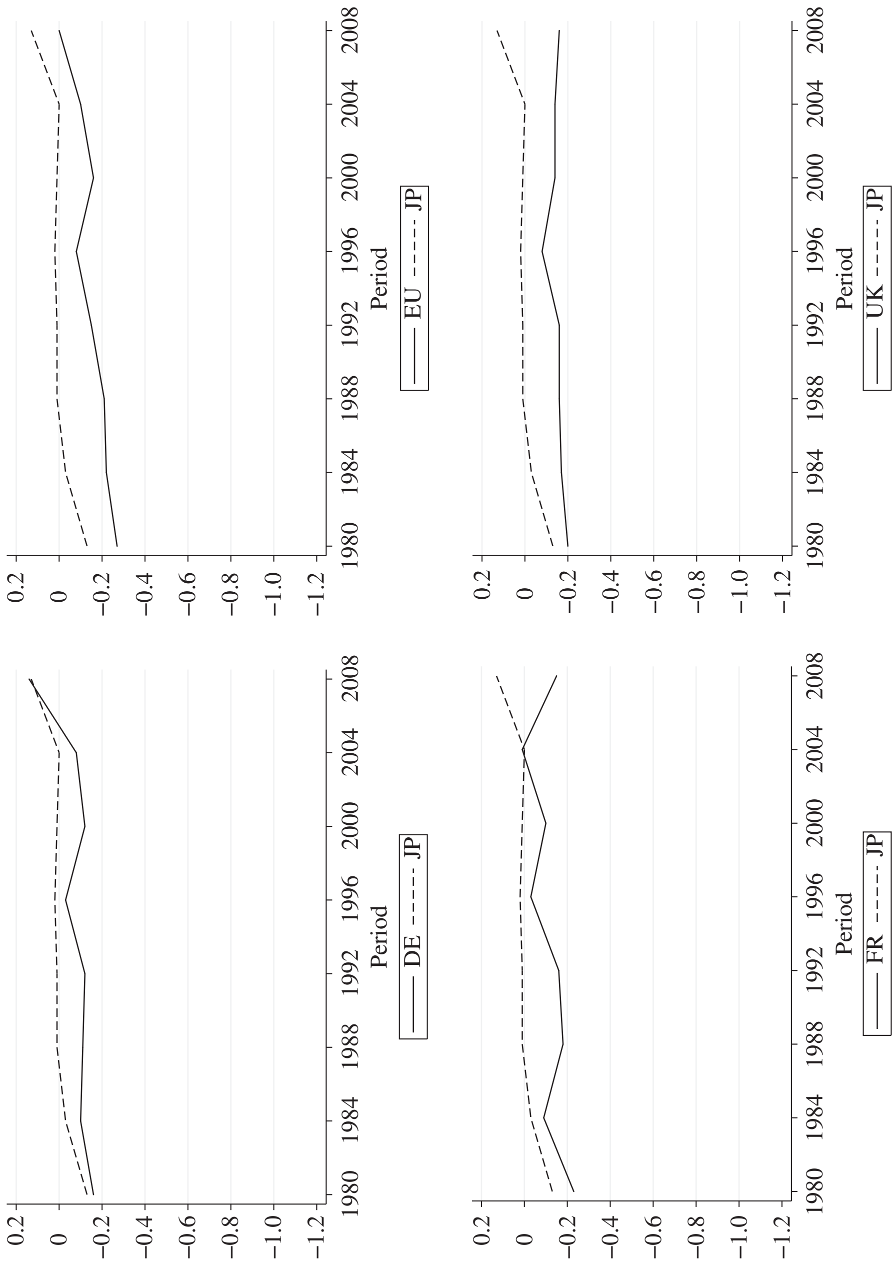

Fig. C2. *Graphical Representation of Estimation Results in Table C10*

Notes. Each graph plots the coefficient values for the country dummy. Japan is included in every graph as a benchmark.

© 2017 The Authors.
The Economic Journal published by John Wiley & Sons Ltd on behalf of Royal Economic Society.

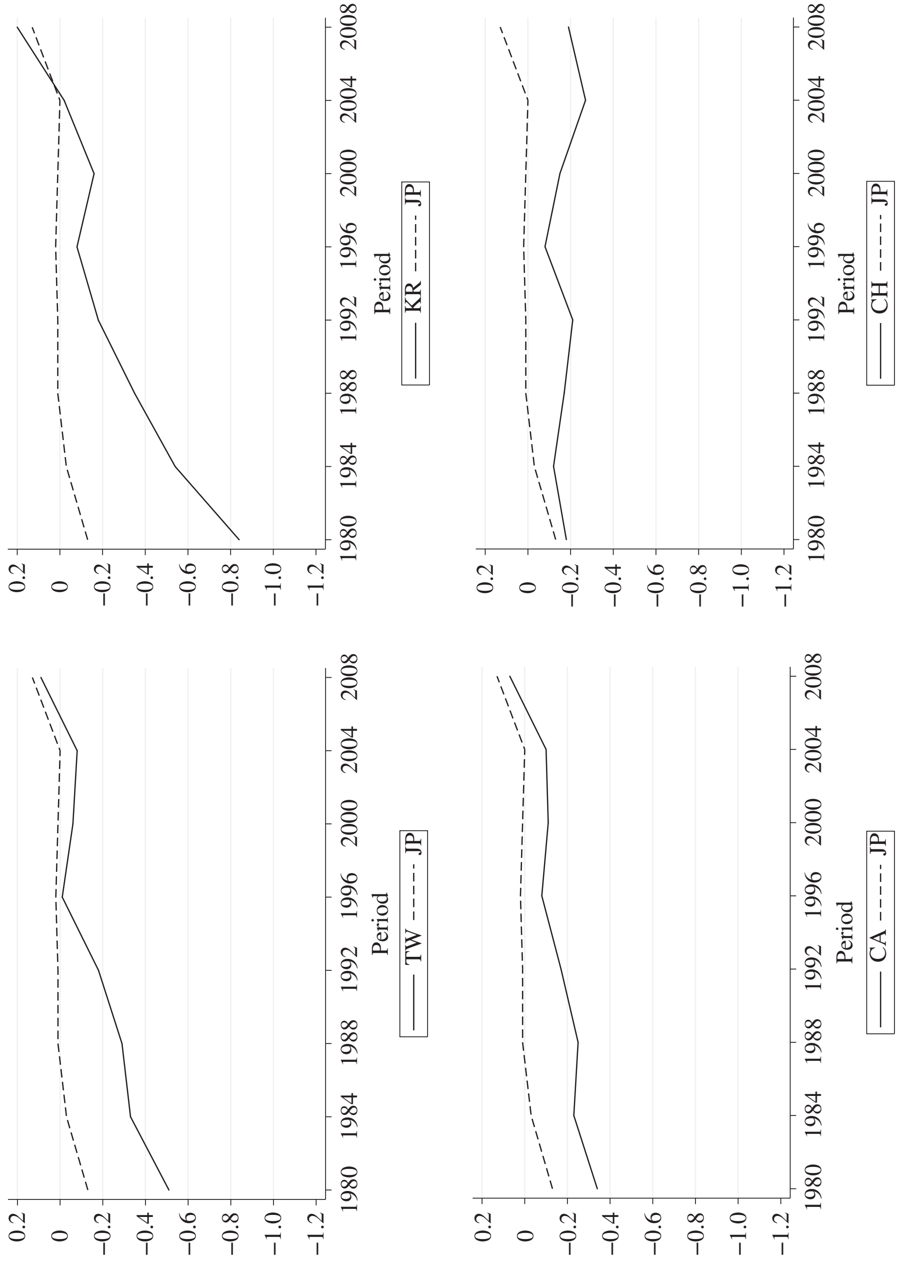

Fig. C2. *(Continued)*

© 2017 The Authors.
The Economic Journal published by John Wiley & Sons Ltd on behalf of Royal Economic Society.

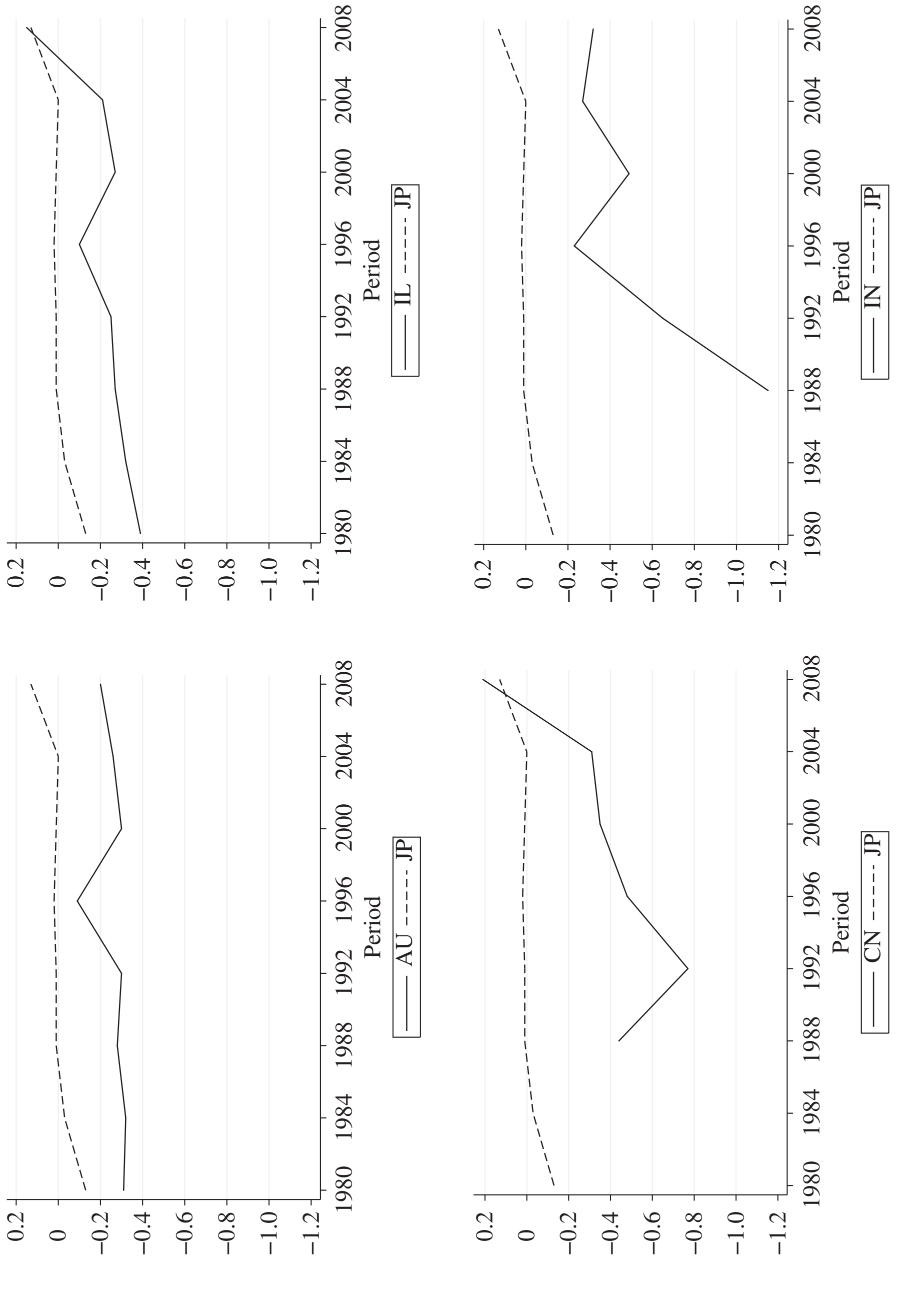

Fig. C2. *(Continued)*

© 2017 The Authors.
The Economic Journal published by John Wiley & Sons Ltd on behalf of Royal Economic Society.

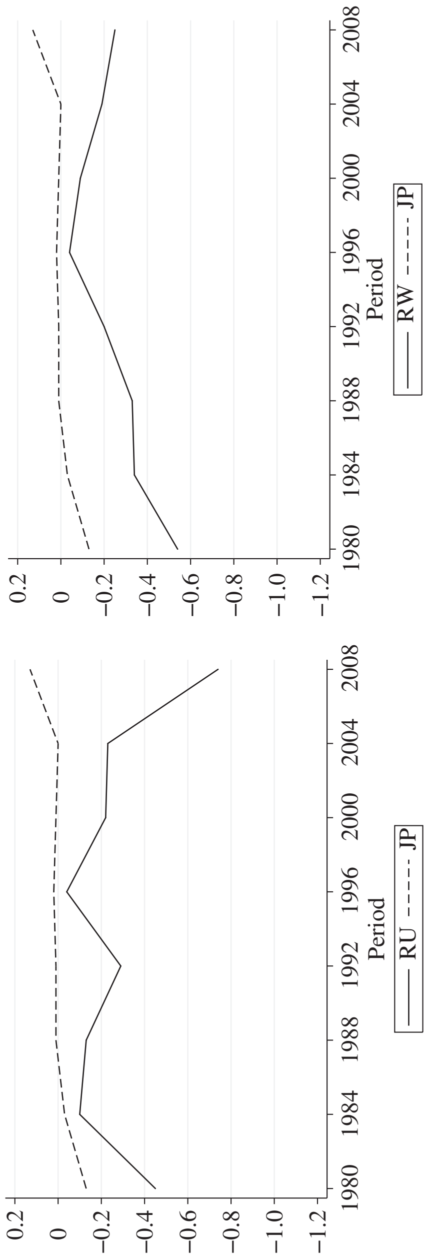

Fig. C2. *(Continued)*

© 2017 The Authors.
The Economic Journal published by John Wiley & Sons Ltd on behalf of Royal Economic Society.

References

Hall, B.H., Jaffe, A.B. and Trajtenberg, M. (2001). 'The NBER patent citation data file: lessons, insights and methodological tools', Working Paper, National Bureau of Economic Research.

© 2017 The Authors.
The Economic Journal published by John Wiley & Sons Ltd on behalf of Royal Economic Society.
